# Hydropeaking strands and displaces larval and juvenile fish across species

**DOI:** 10.1038/s43247-026-03580-2

**Published:** 2026-05-07

**Authors:** Stefan Schmutz, Daniel S. Hayes, Simon Führer, Franz Greimel, Bernhard Zeiringer, Mathias Jungwirth, Stefan Auer

**Affiliations:** Department of Ecosystem Management, Climate and Biodiversity, Institute of Hydrobiology and Aquatic Ecosystem Management, BOKU University, Vienna, Austria

**Keywords:** Environmental impact, Limnology, Hydrology

## Abstract

Hydropeaking, the intermittent operation of hydropower, generates rapid flow fluctuations that disrupt river ecosystems. However, the mechanisms driving stranding and displacement of young fish under varying hydropeaking conditions remain poorly understood. Using a nature-like experimental facility, we conducted ∼1,000 hydropeaking trials involving >120,000 larval and juvenile fish from four species to assess how hydropeaking intensity, environmental conditions, and biotic factors influence fish behavior. Stranding and downstream displacement increased with hydropeaking intensity, particularly at night, in smaller fish, and lower water temperatures. Flow peaks with cold-water releases (thermopeaking) further amplify displacement. Fish demonstrated behavioral adaptation, reducing displacement over successive peaks, suggesting learning effects. The patterns were largely consistent across species. By identifying critical ecological thresholds, our findings inform hydropower strategies that minimize ecological harm while supporting renewable energy production.

## Abstract (web summary)

Hydropeaking is an important renewable energy system but can disrupt river ecosystems and cause ecological harm by stranding and downstream displacing juvenile and larval fish, according to experimental studies on >120,000 fish from four species.

## Introduction

Hydropower is a cornerstone of renewable energy systems, offering operational flexibility to stabilize the variability of wind and solar (PV) power. However, its ecological costs are profound, particularly for riverine ecosystems^1^. Decades of research have documented the impacts of hydropower, including habitat fragmentation, reservoir inundation, water abstraction, altered water quality, and disrupted sediment transport. Yet, the specific effects of hydropeaking (HPK), the practice of adjusting turbine operations to match fluctuations in electricity demand and to optimize revenue, have only recently garnered more attention^2–5^. During periods of low energy demand, water is stored in reservoirs and released during high demand to generate electricity. Around 30–60% of all hydropower facilities may benefit from this operational flexibility^6,7^, including run-of-the-river stations^8^. Given the inherent variability of renewable energy systems, peak-operating hydropower plants will become of even greater importance in future energy systems^9–11^, particularly during dry periods^12^.

However, HPK generates abrupt, artificial flow peaks, often occurring multiple times per day^13^, which propagate downstream and alter hydromorphological processes^1,14–16^. Thermopeaking, short-term temperature fluctuations associated with HPK releases^17–19^, can exacerbate these effects^20–24^. These rapid flow and temperature fluctuations can severely impact riverine organisms and food webs, reducing taxa abundance and diversity, as well as affecting primary productivity up to many hundreds of kilometers downstream of the dam^25–29^.

**Fig. 1:**
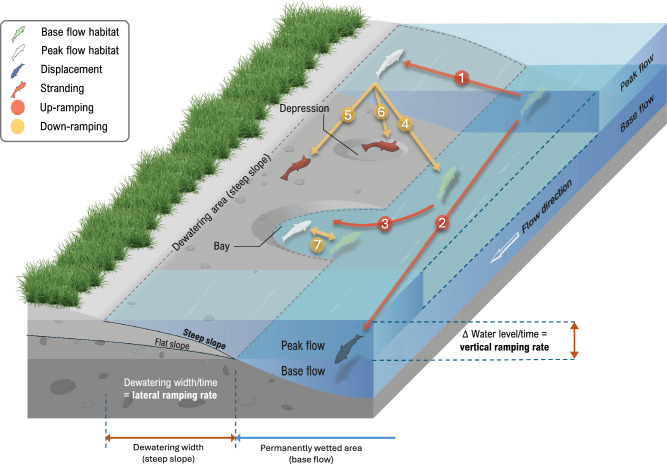
Conceptual framework for the response of young riverine fish to hydropeaking. Larval and juvenile fish prefer shallow, slow-flowing habitats along shorelines. During up-ramping (rising water level), fish (1) follow the shoreline toward the bank to avoid high flow velocities, (2) are displaced downstream, or (3) seek refuge in hydraulic shelters (e.g., bays). During down-ramping (falling water level), fish (4) may find their way back to the permanently wetted area, (5) strand, (6) accumulate in depressions with delayed dewatering and/or increased predation risk, or (7) persist in permanently wetted hydraulic shelters. The response of fish to hydropeaking can depend, in addition to ramping rate, on species, fish length, light conditions, morphology of the dewatered zone, water temperature, and the number of peaks experienced^4,5,10,44^.

Among fish, post-hatch life stages are the most sensitive and vulnerable ones, given their high natural mortality. Hence, the survival of young fish appears to be the most critical bottleneck in mitigating the ecological impacts of HPK^2–4^, while the effects of HPK in other life stages might be lower (e.g., spawning^30^). Juvenile riverine fish predominantly inhabit shallow, slow-flowing areas along riverbanks^31,32^. These habitats are frequently inundated and dewatered during HPK events, exposing young fish to displacement and stranding (Fig. 1). The hydrodynamic changes associated with HPK^33^, especially during rapid up- and down-ramping of flows, can displace fish when hydraulic forces exceed their ability to maintain position. During the up-ramping phase, increasing flow velocities and water depths compel young fish to shift laterally to suitable habitat conditions; otherwise, they risk downstream displacement. Stranding occurs when receding water levels isolate fish, particularly on gently sloped river bars, or in potholes (riverbed depressions) and side channels that become disconnected^34–36^. Beyond displacement and stranding, HPK disturbs fish migration^37^, nesting, and spawning^38^, with nesting sites often dewatered during low-flow periods^39^. Over time, these cumulative impacts can impair the health and resilience of fish communities, which are an indicator for ecosystem integrity^40,41^.

Field studies on HPK are often confounded by natural processes, such as floods, which can obscure its specific effects^42^. Experimental flumes offer a controlled alternative to isolate the abiotic and biotic factors driving fish displacement and stranding. However, traditional indoor flumes (Table S1) are limited by their small scale, constrained water availability, and reduced ecological realism, which limit the transferability of findings to real-world conditions^43^. Recent advancements, such as the outdoor HyTEC facility (hydropeaking.boku.ac.at), have addressed these limitations. The HyTEC facility replicates a natural stream, enabling researchers to simulate realistic gravel bar conditions under HPK flow regimes. This innovative approach bridges the gap between controlled laboratory experimental studies and ecologically relevant field studies, providing critical insights into the impacts of HPK on riverine ecosystems.

One of the most influential factors in fish stranding is the ramping rate, the speed at which water levels recede^44^. Previous field and experimental studies have predominantly focused on salmonid species and have only partially addressed the effects of environmental factors such as water temperature, daylight, and river morphology. Experimental studies on the stranding of juvenile fish remain limited^45–47^, and only recently have researchers begun to study the larval stage, which is likely the most sensitive life stage to HPK disturbances. Similarly, the downstream displacement of fish, or “drift”, has only recently emerged as a focus of HPK research^48–51^, despite its recognition as a critical ecological process^2–4^. Key questions on drift and stranding mechanisms remain unanswered. The fragmented state of current knowledge limits our ability to draw general conclusions about species-specific differences, size-dependent effects, and interactions with environmental factors. This study integrates a decade of research at a near-natural experimental channel facility, enabling the analysis of species-specific differences in displacement and stranding. By analyzing 956 experimental trials with >120,000 larval and juvenile fish of four species (Tables S2and S3) under a consistent study design, this work provides a robust dataset to disentangle the roles of critical factors and their interactions. To this aim, we test the following hypotheses: (i) displacement and stranding of young fish are triggered by specific flow cues; (ii) these processes are influenced by the same environmental (e.g., daylight, water temperature) and biotic factors (e.g., fish length); (iii) there are threshold values of environmental and biotic factors beyond which fish respond significantly to HPK; (iv) different species react in distinct ways to HPK; and (v) juvenile fish may adapt behaviorally to repeated HPK events.

## Methods

### Experiments

All experiments were conducted between 2012 and 2022 at the HyTEC facility (Hydromorphological and Temperature Experimental Channels) in Lunz am See, Lower Austria (47°51'22.4“N, 15°02'12.0“E; hydropeaking.boku.ac.at). The facility comprises two parallel, near-natural experimental channels, each measuring 40 m in length and 6 m in width. The channels can be operated independently, with a maximum discharge of 600 l.s⁻¹ per channel. Discharge rates and their temporal variations can be preprogrammed and controlled separately for each channel (Siemens Simatic S7-30). Flow rates within the pipelines are measured using ultrasonic sensors (Rittmeyer RISONIC 2000; Sensor Type A), while automated gate valves regulate the discharge to achieve the programmed flow conditions.

The water supplied to the facility is derived from Lake Lunz, where it is extracted from two depths: near the surface and approximately 10 m below the mean lake water level. The water is transported to the facility via two pipelines (DN400). At the facility, the pipelines bifurcate, allowing each channel to be supplied with both surface and deep water. This setup enables access to two thermally distinct water sources, facilitating precise control of water temperature in the channels through mixing. The mixing process is carried out in dedicated mixing basins (6 × 4 m) before the water enters the channels.

The flumes feature a longitudinal slope of 0.5% and a one-sided riverbank with microlithal substrate (*d*_50_ = 10 mm; *d*_90_ = 21 mm). At the downstream end of each flume, the water flows over a sill into removable, horizontally mounted mesh frames to capture drifting fish. Experiments were conducted either in the entire flumes (*N* = 598) or—due to the vulnerability of the small cyprinid larvae—in mesocosms (*N* = 359). Because of the different spatial scales, experimental designs differed slightly between the flumes and mesocosms, flume experiments either included up- and down-ramping phases to analyze displacement and stranding (*N* = 278) or focused only on down-ramping to quantify stranding (*N* = 311). Mesocosm experiments only tested down-ramping (for more details, see Supplementary Materials^49^ and refs. ^52,53^).

### Data characteristics

The experiments were designed to investigate the proportion of displaced and stranded fish (%)^49^ as functions of HPK variables, i.e., vertical (RampRate) and lateral ramping rates (RampLat), peaking frequency (MultPeak), flow amplitude (Ampl), flow ratio (flowRatio), peak duration (PeakDur), fish species, and fish length (Length), while also considering additional factors such as daylight (DayNight), channel morphology, i.e., gravel bar (MorphBar), bar slope (BarSlope), bay (MorphBay), depression (MorphDepression), and water temperature (Temp) (Table S2 and S3). Vertical ramping rates ranged from 0 cm min⁻¹ (control) to 3 cm min⁻¹, reflecting typical rates of change observed in Alpine rivers impacted by HPK^40^.

Four dominant fish species from rhithral and epipotamal river regions were tested: two salmonids—brown trout (*Salmo trutta*, *N* = 186) and European grayling (*Thymallus thymallus*, *N* = 364)—and two cyprinids—nase (*Chondrostoma nasus*, *N* = 344) and barbel (*Barbus barbus*, *N* = 63). We selected these species because they bracket key ecological traits and life histories relevant to HPK responses and management in Alpine rivers. Brown trout and European grayling are cold-water, rheophilic, lithophilic species; brown trout is demersal and typically remains close to the substrate (station-holding)^54^, with grayling juveniles sometimes schooling^55–57^. Whereas brown trout is more structure-associated, European grayling more often occupies open water^58^. Nase and barbel are rheophilic, lithophilic cyprinids of cool- to warm-water guilds that spawn in late spring–early summer^58^; their larvae rapidly drift to and occupy shallow, low-velocity shorelines and frequently shoal^59^. All four species use gravel bars and margins during early life stages—habitats most prone to rapid dewatering—making them especially relevant for HPK impact assessment. A priori, we expected species-specific differences in displacement and stranding driven by differences in body size at emergence, temperature preferences, and behavior. Including four species from two families therefore provided a management-relevant test of whether species identity adds explanatory power beyond body size, diel period, and temperature. Fish lengths ranged from 12.4 to 70 mm, encompassing both larval and juvenile stages. Each experiment used fish of similar size; we therefore assigned the batch mean length to each experiment.

Experiments were conducted during both day (*N* = 611) and night (*N* = 346). Channel morphology was manipulated by varying gravel bar slopes (2–11%) and habitat features^51^, including: (a) flat gravel bars (*N* = 659), (b) gravel bars with depressions (*N* = 276), and (c) gravel bars with bays (*N* = 22). Water temperature was held constant during each experiment, ranging from 3 to 21 °C, except during thermopeaking experiments, in which temperature was increased (ThermoPeakHot) or decreased (ThermoPeakCold) during peaking events^18,19,50^. A subset of experiments (*N* = 104) included up to nine consecutive peaks^60^ to assess the effects of repeated HPK^13^ (Tables S2and S3).

### Statistical analysis

The primary objective of the experiments was to identify thresholds for the onset of fish displacement and stranding. Consequently, most experiments were conducted near the expected ramping rate thresholds, resulting in a skewed dataset with a high concentration of data points around these thresholds. To address this imbalance, the Synthetic Minority Oversampling Technique (SMOTE) algorithm was applied^61^. Using the R package *smotefamily*, (a) new samples for minority classes were generated using the nearest neighbor method, and (b) subsamples were taken from the majority classes to achieve a balanced distribution among stranding classes (0, 5, 12.5, 25, 50, 75, 100). Models trained on balanced data performed well across the full range of the target variable, while models trained on unbalanced data tended to underestimate high stranding and displacement values (Fig. S2).

Correlations among predictor variables were assessed using Spearman’s rank correlation. HPK variables with ∣r∣>0.6 (lateral ramping rate, flow ratio, flow amplitude, and base flow) were excluded (Table S2). Among the remaining non-HPK variables, correlations were <0.7, so no further exclusions were necessary. Random forest modeling was selected to analyze fish responses to HPK using the R package *randomForest*^62^. Random forests were chosen for their robustness to overfitting, ability to capture non-linear relationships and higher-order interactions, and inherent variable selection capabilities^62,63^. These features make random forests particularly well-suited for analyzing complex datasets from multiple experimental studies, as they can account for specific experimental conditions while also identifying overarching effects across diverse scenarios.

Model parameters were optimized for the “number of variables used for node splits” (tested values: *N* = 3, 5, 9) and the “minimum number of samples per node” (tested values: *N* = 5, 10, 20, 30) using bootstrapping with the R package *caret*. Optimizing random forest parameters recommended using five variables per split and a minimum node size of 10 samples (Fig. S3). A predefined number of trees (*N* = 500) was grown, and 10-fold cross-validation was applied to ensure robust model performance.

Model performance was evaluated using mean squared error (MSE) and goodness of fit (*R*²) (Table S4). To further assess model fit and the distribution of predicted values, observed values were plotted against predicted values (Fig. S2). Three separate models were developed for both stranding and displacement to: (a) distinguish between HPK and other effects, and (b) evaluate the impact of balancing the dataset (Table S4).

Variable importance was evaluated by measuring the percentage increase in MSE when individual variables were shuffled, using the *importance* function from the *randomForest* package. To visualize the marginal effect of individual predictors on the model’s predictions while holding all other variables constant, partial dependence plots were generated with the *partialPlot* function (Figs. S6 and S7).

Interactions between variables were explicitly accounted for in the random forest models, where variables are sequentially split within the tree hierarchy. The importance of two-way interactions was quantified by calculating the level of occurrence (mean minimum depth) of splits for the five most important variables, using the *min_depth_interactions* function from the *randomForestExplainer* package. Interactions between lateral ramping rate, vertical ramping rate, and bar slope were analyzed by variance partitioning using the lateral ramping rate as independent variable and adding vertical ramping rate, bar slope, and their interaction as dependent variables in a stepwise approach.

To identify thresholds—marginal values where small changes in a feature result in a significant change in the target variable—we calculated the mean split values at the first and second to fifth tree levels for numeric variables (Figs. S4 and S5). These thresholds were subsequently used to develop predictive scenarios (see below).

To further analyze and visualize the relationships of individual effects and their interactions to model predictions, we applied the SHAP (SHapley Additive exPlanations) methodology using the *fastshap* package in R. SHAP provides a consistent and interpretable framework for understanding the contribution of each predictor to the model’s output, enabling a deeper understanding of the underlying relationships^64–66^. The SHAP method is a tool for explaining the output of machine learning models by fairly distributing the model’s prediction among the input features. The sum of the SHAP values for all features equals the difference between the model’s prediction for a specific instance and the average prediction across all instances. Each feature’s contribution is fairly allocated based on its marginal contribution across all possible subsets of features (Figs. S8 and S9).

To assess the potential influence of experimental settings on the model results, we tested correlations between model residuals and variables representing experimental settings. These included channel side (left or right), stocking density, and spatial dimension (full channel or mesocosm). Spatial dimension was only tested for stranding experiments, as all displacement experiments were conducted in the full channel. No significant correlations were observed between model residuals and variables representing experimental settings (channel side, stocking density, and spatial dimension) (Table S6).

Finally, the models were used to simulate scenarios based on the identified thresholds. Predictor variables were averaged for subsets of data below and above the thresholds and then used as model inputs. These simulations illustrate the effects of HPK gradients on fish under biologically (e.g., species, body length) and environmentally limiting conditions (e.g., diel period, temperature). All analyses were conducted in R^©^ (version 4.4.3)^67^.

#### Diversity, equity, ethics and inclusions

Animal Welfare Statement: We fully committed to adhering to the highest standards of animal welfare, in line with the principles and requirements of the EU Directive 2010/63/EU and its national implementation in Austria (Tierversuchsgesetz 2012, TVG 2012) on the protection of animals used for scientific purposes. All animals under our care, including fish, were treated with respect and provided with conditions that meet or exceed the standards set by the Directive. In our work, we strictly followed the 3 R principles. The HyTEC facility is designed to provide species-appropriate housing, optimal water quality, and proper nutrition, ensuring the animals’ physiological and behavioral needs are met. Permanent monitoring and care were provided by trained personnel to ensure the well-being of the fish at all times. While we fully complied with the welfare standards outlined in the Directive and national legislation, no formal/additional project authorization or reporting to the authorities has been undertaken. This is because the nature of our work does not inherently involve procedures that would necessarily cause pain, suffering, or death. Stranded or drifted fish are immediately recovered from the experimental channel and taken back to the holding tanks. As such, we operate under the understanding that the Directive’s requirements and national legislation for authorization and reporting are not applicable in this specific context.

### Reporting summary

Further information on research design is available in the Nature Portfolio Reporting Summary linked to this article.

## Results and discussion

### Lowering hydro- and thermopeaking intensity safeguards fish

Our replicated and controlled experimental study design allowed us to quantify fish displacement and stranding under near-natural conditions as a response to HPK and environmental factors. We conducted HPK experiments with larval and juvenile stages of brown trout (*Salmo trutta*), European grayling (*Thymallus thymallus*), nase (*Chondrostoma nasus*) and barbel (*Barbus barbus*). The developed models explain 88% of the variation in both stranding and displacement, with most of the variation attributed to HPK (69% for stranding and 66% for displacement; Table S4). HPK intensity is quantified by the following variables selected by the model: Ramping rate (cm min⁻¹)—the speed of water level change (up-ramping equals down-ramping rate, RampRate), peak duration (min)—the duration of constant peak flow (PeakDur), peak flow (l s⁻¹)—the flow magnitude during the peak phase (PeakFlow), the number (N) of multiple consecutive peaks (MultPeak) and cold thermopeaking (ThermoPeakCold)—lowered water temperature during peaking events^19^. Thermopeaking refers to the combination of flow peaks and short-term alterations in water temperature, both caused by HPK. This phenomenon occurs when water is released by reservoirs from deepwater layers with different water temperature than the river. As a result, thermopeaking can cause cooling during summer and warming during winter^19^, leading to declines in growth and survival of juvenile fish^68^.

**Fig. 2:**
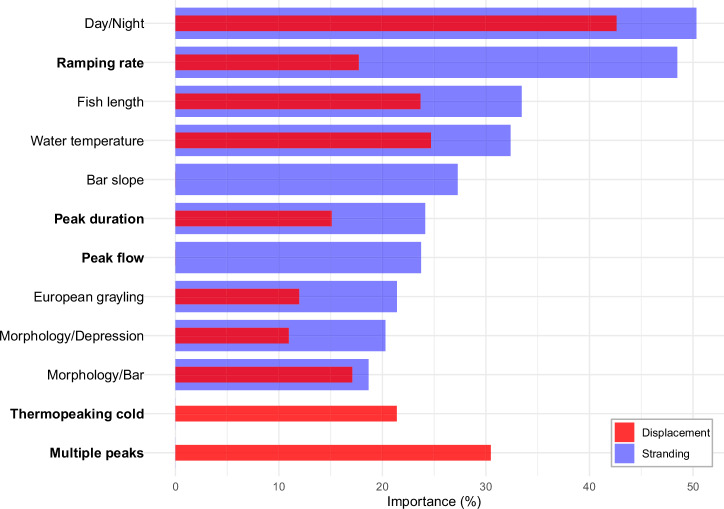
Variable importance for displacement and stranding. The top 10 variables are shown for each response. Importance is calculated as the mean squared error increase when individual variables are shuffled (see “Methods”). Hydropeaking variables are shown in bold.

**Fig. 3:**
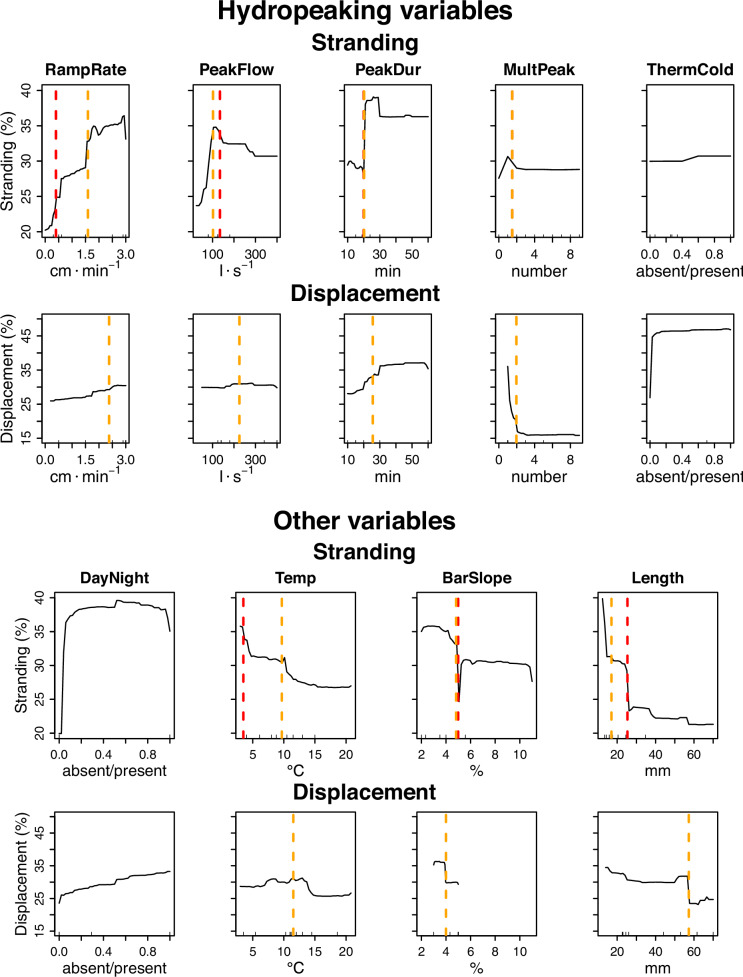
Partial dependence plots for hydropeaking (top) and other important variables (bottom) from the random forest models. Vertical lines indicate median split thresholds for numeric variables (Table S5, and Figs. S4, S5) used by the trees (red: first split level, orange: levels 2–5). Only the most important variables are shown (see Figs. S6 and S7 for the full set of partial dependence plots).

The most influential HPK variable for displacement is the number of consecutive peaks. Displacement decreases sharply after the first peak and remains consistently low through the subsequent peaking events, regardless of fish length. Cold thermopeaking, higher ramping rates, and longer peak durations also contribute to increased displacement, although thresholds for these variables are less distinct (Figs. 2, 3 and Table S5). In our experiments, a ∼3 °C decrease in water temperature coupled with HPK resulted in a ∼20% increase in drift. Rapid decreases in water temperature can induce “cold shock” in fish, leading to reduced swimming performance^69^. Acute exposure to cold water has been shown to severely impair metabolic recovery following exhaustive exercise in Atlantic salmon (*Salmo salar*)^70^. A cold shock, combined with increased hydraulic stress during HPK, may further amplify downstream drift. The results suggest that larvae respond more strongly to temperature changes than to hydraulic conditions, as has been described for nase^48^ and European grayling^50^.

Stranding rates exhibit a positive relationship with HPK intensity increases, with ramping rate identified as the most critical HPK factor influencing stranding (Fig. 2). Thresholds were observed at ramping rates of 0.4 cm min⁻¹ and 1.6 cm min⁻¹ (Figs. 3, S4 and Table S5). Our findings confirm previous studies showing that stranding is strongly related to down-ramping rates^45–47^ (Table S1). Even slow down-ramping (<0.4 cm min⁻¹) results in stranding rates of 20–45% for larval fish during a single event, with higher ramping rates increasing stranding rates to ∼60% (Fig. 3). The lower stranding rates reported in earlier indoor experiments (Table S1, ≤22%) may be explained by the use of larger fish (40–90 mm) compared to the smaller fish in our experiments (mean: 25 mm). However, under cold winter conditions, stranding rates in indoor studies also have been reported to reach up to 90%^45^ (Table S1).

Comparing our results with field studies is challenging due to the inherent difficulties in quantifying stranding and drift in natural environments^35,36,40,71,72^. Additionally, environmental conditions in the field are more variable and often insufficiently monitored. Nevertheless, the use of alternative metrics, such as biotic indices, may allow for indirect comparisons. For example, a study on Austrian rivers^40^ indicated that fish stranding caused by ramping rates >0.25 cm min⁻¹ is likely a major driver of fish community degradation, particularly when such events occur more than 20 times per year.

Among HPK variables, peak flow and peak duration were less influential than ramping rate in our stranding model, with thresholds of 100–130 l s⁻¹ and 20 min, respectively (Fig. 3). A peak flow of 115 l s⁻¹ in our experimental facility corresponds to a shift of the waterline of about 0.65 m and 1.9 m at steep (11%) and flat (2%) gravel bars, respectively – values that are often exceeded in hydropeaked rivers^72^. Additionally, stranding slightly decreases after the first and subsequent consecutive HPK events, suggesting a potential adaptation effect. Indeed, a study on brown trout found that fry were stranded twice as often during the first dewatering episode compared to subsequent episodes (second to fifth)^47^. However, the fish used in that study were significantly larger (56–88 mm), had been used multiple times for experiments, and, consequently, appear to be more “experienced” than those used in our experiments^47^. Even though fish used in our multipeaking experiments had similar body lengths, size-selective effects in other contexts cannot be ruled out. Finally, the model indicates that cold thermopeaking leads to a slight increase in stranding (Fig. 3).

### Environmental factors amplify hydropeaking effects

Even though our models’ variability is largely attributed to HPK effects (Table S4), also other environmental parameters, including time of day, ambient water temperature, bar slope, and morphological river bar configuration, are also key (Fig. 2).

Our results show that downstream displacement significantly increases during the nighttime under HPK conditions (Fig. 3), likely exacerbating the well-documented behavior for larval fish under undisturbed flow conditions^55–57^. Nighttime was found to have a greater influence on stranding than ramping rate or other individual HPK factors (Fig. 3). Stranding rates were approximately twice as high during nighttime compared to daytime conditions. This may be due to the behavior of larvae, which tend to occupy shallow areas near the riverbank at night and move further offshore during the day^31,32^. This finding aligns with, but also partially contradicts, other indoor studies on salmonids, which suggest that seasonal habitat preferences may play a role. In spring and summer, young fish often use open water or above-substrate habitats, whereas in winter, they hide within the substrate during the day, increasing their risk of stranding^45–47^.

Ambient water temperature seems to play a minor role in controlling drift (Fig. 3), except for cold thermopeaking (see above). However, low water temperatures significantly increased stranding, with thresholds identified at 3.5 °C and 9.7 °C (Figs. 3, S4 and Table S5). Under cold conditions, stranding rates are up to ~10% higher compared to warm conditions. Temperatures below 3.5 °C, which predominantly occur in winter, lead to the stranding of larger fish, whereas larval fish are more affected by stranding in spring when the temperatures are ~10 °C (Fig. 5).

The slope of the gravel bar also played a role, with flat slopes of <5% resulting in higher displacement and stranding compared to steeper slopes (≥5%), a finding which is consistent with results from indoor experiments on salmonids^45^ (Table S1). Morphological configurations, such as gravel bars and depressions, contributed to both stranding and displacement; however, their effects were relatively minor compared to other factors (Fig. 2). Depressions (potholes) slightly increase stranding (Fig. S6), as fish tend to accumulate in these areas during the retreat of the water and ultimately become trapped without the possibility of escape^35,49,71^.

### Size trumps species

We anticipated varying responses to HPK among the tested species, as they belong to different families (salmonids and cyprinids) with distinct temperature and habitat preferences. Critical swimming speed, which is crucial for withstanding the increased velocities caused by HPK, is influenced by both fish size and water temperature^73^. We expected lower displacement and stranding rates for salmonids, as they have larger larvae and are better adapted to cold water compared to cyprinids. However, the differences among species were only marginal, playing a minor role compared to other factors previously described. Compared to cyprinids, brown trout and European grayling exhibited slightly higher stranding rates, with the latter also showing higher drift rates than the other species (Figs. 2, S6 and S7). One possible explanation is that, despite being smaller, cyprinids tend to prefer open water^59^, whereas salmonids remain closer to the substrate^45^. Other experiments have shown that coho salmon (*Onchorynchus kisutch*) are more likely to be stranded than rainbow trout (*O. mykiss*), although the latter have been larger, making direct comparisons challenging^45^. The shoaling behavior of cyprinids^59^ may also increase the chances of escaping stranding^51^, although this has been documented for European grayling as well^31^. A recent field study found no interspecific differences between salmonid and cyprinid species: similar HPK events induced “salmonid fry” stranding events and “super-stranding” events (massive stranding of many taxa, mainly cyprinids), primarily associated with the presence of scour pool microstructures; however, stranding was only monitored during the day in this study^35^. It appears that environmental factors such as daylight, morphological trapping structures, and water temperature often outweigh species-specific differences in the response of early life stages to HPK, however, further investigations are necessary to confirm this assumption.

Size-dependent effects on displacement are less pronounced, with only a slight reduction in displacement observed for fish >57 mm. It is known that downstream drift becomes less significant in the post-larval phase even under undisturbed hydrological conditions^57,74^. In contrast, stranding rates for smaller fish (<17 mm) are approximately twice as high as those for larger fish (>25 mm) (Figs. 3, S6 and Table S5). Previous indoor experiments are not comparable as they used larger fish than we did (Table S1). Considering the sensitivity of small (larval) fish to HPK will be essential in formulating effective mitigation strategies to avoid losses through stranding^75^.

### Interactions and cumulative effects

**Fig. 4:**
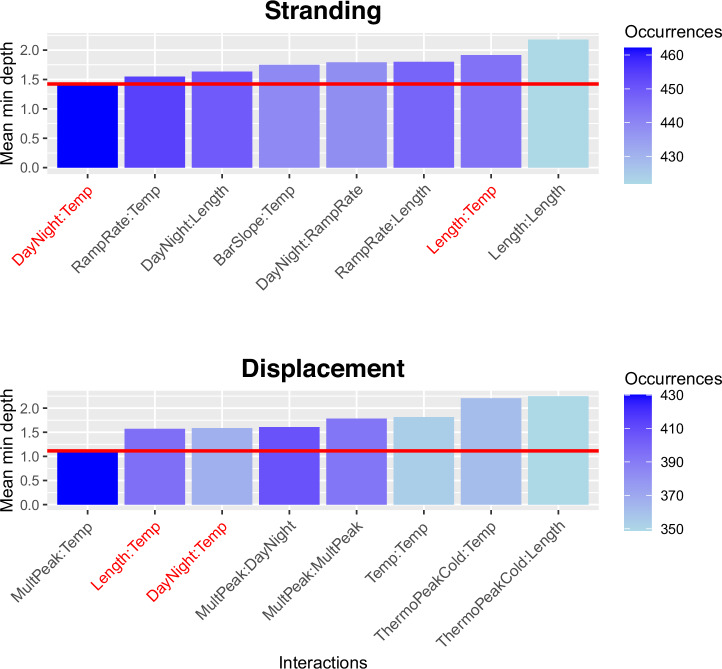
Interactions among predictor variables for stranding (top) and displacement (bottom). Interactions are calculated based on their frequency across random forest tree hierarchies, assigning higher importance to interactions at lower split depths and lower importance to those at higher split depths (i.e., lower vs. higher mean minimum depth). “Occurrences” denotes the total number of interactions across all trees (*n* = 500). Interactions in red indicate those common to both stranding and displacement.

**Fig. 5:**
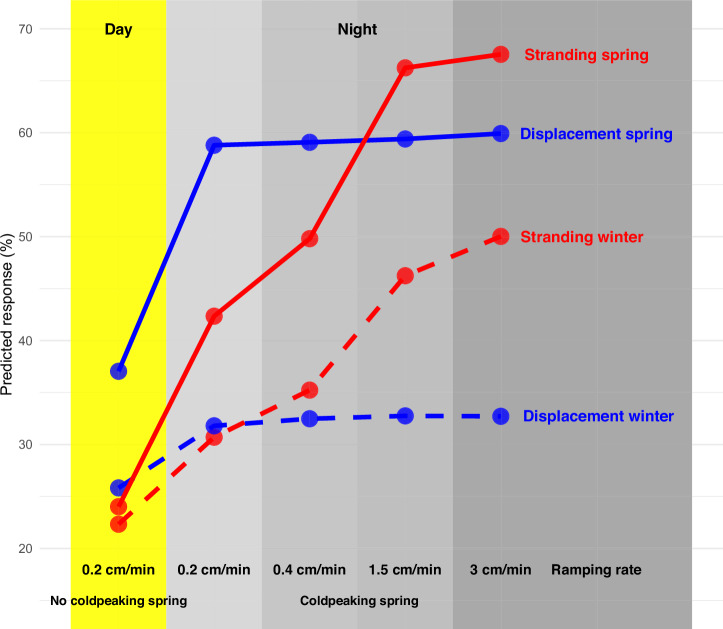
Scenarios for stranding (red) and displacement (blue) responses to hydropeaking depending on ramping rate, diel period (day vs. night), and thermopeaking for a single peaking event. Responses are predicted using the developed random forest models and by holding other factors constant, set below/above identified thresholds, representing realistic environmental conditions for larval fish in spring (solid line) and juveniles in winter (dashed line) on a gravel bar (fish length: spring 14.1 mm, winter 60 mm; temperature: spring 10 °C, winter 3 °C; bar slope = 3%; peak duration = 30 min; peak flow = 400 l s⁻¹, non-species specific). Cold thermopeaking is only relevant in spring.

Incorporating environmental and biological factors into the analysis increases the explained variation in the experiments to as much as 88% for both displacement and stranding (Table S4). This increase can be attributed to both (a) the combined effects of HPK, environmental, and biological factors, and (b) the interactions among these factors (Fig. 4). For instance, conditions such as low water temperatures, small fish size, nighttime, high ramping rates, and flat bar slopes collectively result in significantly higher stranding rates compared to their opposite conditions (Figs. 5 and S8).

Displacement is primarily triggered by the first of multiple peaks and cold thermopeaking. However, when combined with factors such as small fish size, low water temperatures, and nighttime conditions, displacement rates are further elevated compared to their opposite conditions (Figs. 5 and S9). In detail, cold thermopeaking nearly doubles, while multiple peaks (after the first one) halve the proportion of displaced fish under HPK conditions (Fig. 3). These phenomena suggest that larval responses to HPK are more active than passive, in contrast to processes such as plant propagule transport^76^. It appears that larvae use drift as a strategy to avoid cold water, but are also capable of learning that, after withstanding the first peak, maintaining their position is a successful survival strategy during subsequent peaks^13^.

While most studies have focused on the vertical ramping rate, the lateral retreat of the waterline—referred to as the lateral ramping rate—may be more relevant for fish, particularly in the context of large gravel bars^16,44^. In such cases, the risk of stranding increases with the distance fish must traverse to follow the receding water. As expected, vertical and lateral ramping rates are linked by the gravel bar slope, highlighting the interconnected nature of these factors^77^. Variance partitioning analysis of our data reveals that (a) the lateral ramping rate accounts for 64.4% of the variation in the vertical ramping rate, (b) the slope contributes an additional 16.8%, and (c) the interaction between these two factors explains a further 7.9%, resulting in a total of 89% of the variation explained. These findings suggest that using the lateral ramping rate as a more integrative parameter could enhance the accuracy and applicability of future HPK studies^72^.

Since stranding and displacement exhibit similar responses to HPK, it is not surprising that they are significantly correlated (*r* = 0.623, *p* < 0.001). Interestingly, even after controlling for the influence of independent variables, a significant correlation remains (*r* = 0.26, *p* < 0.001), suggesting an intrinsic link between these two processes. Consequently, as suggested above, displacement may be better characterized as active drift rather than passive displacement. Detailed displacement analyses of a subset of the data revealed that displacement primarily occurs during the up-ramping and peak phases, rather than during the down-ramping phase^49^. This aligns with our findings of increased drift during the first 30 min of peaking events (Fig. 3). Such behavior may indicate that displacement serves as a strategy to avoid unfavorable conditions and, consequently, reduce the risk of stranding (Fig. 1). It appears that the more fish are stressed and prone to stranding, the more likely they are to drift downstream, or vice versa. This highlights the need for individual-based experiments to investigate the fate of (non-)drifted and (non-)stranded fish in detail.

Even low drift rates during multiple peaks can lead to a thinning of local fish density^13^. Fish that drift downstream may be preyed upon^78^, encounter habitat conditions outside their preferred range, or face degraded habitat conditions in downstream river sections^79^, potentially resulting in reduced overall recruitment success^80^. In addition to these effects, recruitment in hydropeaked rivers can also be affected by disturbance of spawning^30,38^, displacement and mortality of eggs (through drying or detachment from the substrate)^39,81^ and mortality of pre-gravel emergence life stages (through dewatering)^75,82^.

## Conclusions

Our controlled experimental study design in a nature-like outdoor environmental flume disentangles the effects of HPK as well as environmental and biological parameters on the displacement and stranding of larval and juvenile fish. Our results show that displacement and stranding are triggered by specific flow cues, particularly associated with ramping rate, peak flow, duration, and frequency, supporting our hypotheses. While the ramping rate has traditionally been regarded as the most critical factor influencing the stranding of young fish, our results demonstrate that the extent of fish responses is also strongly influenced by other biological and environmental factors, for both displacement and stranding (supporting our hypotheses), including diel period, fish length, water temperature (and its changes), and river bar morphology. Observed effects may be associated to changes in the swimming ability and activity level of fish under varying environmental conditions, as is also known for fish in other contexts^83^. Therefore, focusing solely on ramping rates may provide an incomplete understanding, particularly when developing mitigation strategies and regulations aimed at improving ecological conditions in HPK-affected rivers. In developing such mitigation rules, it is crucial to consider the appropriate temporal scale of measurements when assessing stranding risks. Stranding occurs within minutes; therefore, measuring peaking events over intervals of half an hour or more may lead to wrong conclusions, as down-ramping rates measured below hydropower plants rapidly change over short time scales.

While our data did not confirm species-specific differences, contrary to our assumptions, we could detect threshold values for environmental and biological factors beyond which fish respond significantly to HPK, supporting our hypotheses. Synthesizing findings of our experiments within realistic spring and winter scenarios reveals a higher risk of displacement and stranding during spring compared to winter. This increased risk is primarily associated with the smaller fish sizes present in spring. Even low ramping rates (~0.2 cm min⁻¹) significantly elevate displacement and stranding during nighttime, particularly when combined with cold thermopeaking (Fig. 5).

One could argue that hydropeaks are analogous to natural events such as floods, to which biota are evolutionarily adapted. However, while natural floods also cause drift and stranding, they are rare compared to hydropeaks, which occur multiple times a day and exhibit faster ramping rates than natural flow fluctuation events. Although we observe behavioral adaptations to multiple peaks in terms of drift—evidenced by a significant reduction in displacement after the first peak—stranding rates remain largely unchanged compared to single peaks, providing partial support for our hypotheses. The cumulative effect of stranding from multiple hydropeaks occurring within a few days during the critical larval phase could rapidly extirpate an entire new generation of fish for that year.

Hydropower is a renewable energy source, but its sustainability is constrained by its impacts on aquatic ecosystems, including HPK. This study advances our understanding of the processes behind larval and juvenile fish displacement and stranding. Such knowledge enables the optimization of flexible hydropower production to enhance ecological integrity while meeting societal energy demands.

## Supplementary information

Transparent Peer Review file

Supplementary Information

Reporting summary

## Data Availability

All data used in the analysis^84^ are publicly available at 10.5281/zenodo.19661575.

## Code availability

The computer code used in the analysis^84^ is publicly available at 10.5281/zenodo.19661575.

## References

[1] He, F. et al. Hydropower impacts on riverine biodiversity. *Nat. Rev. Earth Environ.* **5**, 755–772 (2024).

[2] Bipa, N. J., Stradiotti, G., Righetti, M. & Pisaturo, G. R. Impacts of hydropeaking: a systematic review. *Sci. Total Environ.* **912**, 169251 (2024). 38101637 10.1016/j.scitotenv.2023.169251

[3] Bozeman, B. B., Pracheil, B. M. & Matson, P. G. The environmental impact of hydropower: a systematic review of the ecological effects of sub-daily flow variability on riverine fish. *Rev. Fish Biol. Fish.* **35**, 45–76 (2025).

[4] Young, P. S., Cech, J. J. & Thompson, L. C. Hydropower-related pulsed-flow impacts on stream fishes: a brief review, conceptual model, knowledge gaps, and research needs. *Rev. Fish Biol. Fish.* **21**, 713–731 (2011).

[5] Smokorowski, K. E. The ups and downs of hydropeaking: a Canadian perspective on the need for, and ecological costs of, peaking hydropower production. *Hydrobiologia* **849**, 421–441 (2022).

[6] Jardim, P. F. & Collischonn, W. Sub-daily flow alterations (hydropeaking) due to reservoir operations in Brazil. *Rev. Bras. Recur. Hidricos* **29**, 10.1590/2318-0331.292420230111 (2024).

[7] Johnson, M. M., Kao, S.-C. & Uría-Martínez, R. *Existing Hydropower Assets (EHA) Plant Database*. 10.21951/EHA_FY2025/2568751 (2025).

[8] Almeida, R. M. et al. Hydropeaking operations of two run-of-river mega-dams alter downstream hydrology of the largest Amazon tributary. *Front. Environ. Sci*. **8**, 120(2020).

[9] Marttila, H.et al.River systems under peaked stress. *Environ. Res. Lett*. **19**, 064071 (2024).

[10] Hayes, D. S. et al. Hydropeaking: Processes, Effects, and Mitigation. In *Encyclopedia of Inland Waters, Second Edition* **4**, 134–149 (Elsevier Inc., 2022).

[11] Ashraf, F. B. et al. Changes in short term river flow regulation and hydropeaking in Nordic rivers. *Sci. Rep.* **8**, 17232 (2018). 30467316 10.1038/s41598-018-35406-3 PMC6250702

[12] Déry, S. J., Hernández-Henríquez, M. A., Stadnyk, T. A. & Troy, T. J. Vanishing weekly hydropeaking cycles in American and Canadian rivers. *Nat. Commun.* **12**, 1–12 (2021). 34887399 10.1038/s41467-021-27465-4 PMC8660798

[13] Hayes, D. S. et al. Why hydropeaking frequency matters: effects of recurring stranding on fish. *J. Ecohydraulics* **10**, 302–318 (2024).

[14] Greimel, F. et al. PeakTrace: routing of hydropeaking waves using multiple hydrographs—A novel approach. *River Res. Appl.* **39**, 326–339 (2023).

[15] Batalla, R. J. et al. Hydropeaked rivers need attention. *Environ. Res. Lett*. **16**, 021001 (2021).

[16] Hauer, C., Holzapfel, P., Leitner, P. & Graf, W. Longitudinal assessment of hydropeaking impacts on various scales for an improved process understanding and the design of mitigation measures. *Sci. Total Environ.* **575**, 1503–1514 (2017). 28346992 10.1016/j.scitotenv.2016.10.031

[17] Zolezzi, G., Siviglia, A., Toffolon, M. & Maiolini, B. Thermopeaking in Alpine streams: event characterization and time scales. *Ecohydrology* **4**, 564–576 (2010).

[18] Toffolon, M., Siviglia, A. & Zolezzi, G. Thermal wave dynamics in rivers affected by hydropeaking. *Water Resour. Res.* **46**, 1–18 (2010).

[19] Zolezzi, G., Siviglia, A., Toffolon, M. & Maiolini, B. Thermopeaking in Alpine streams: event characterization and time scales. *Ecohydrology* **4**, 564–576 (2011).

[20] Bruno, M. C., Silveri, L. & Maiolini, B. Studies on the impacts of hydropeaking on hyporheic invertebrates of an Alpine stream. *Geophys. Res. Abstr*. **12**, European Geosciences Union General Assembly 2010. Vienna, Austria, 02 – 07 May 2010. url: http://meetingorganizer.copernicus.org/EGU2010/EGU2010-10001.pdf handle: http://hdl.handle.net/10449/21725, accessed 4 May 2026.

[21] Bondar-Kunze, E., Maier, S., Schönauer, D., Bahl, N. & Hein, T. Antagonistic and synergistic effects on a stream periphyton community under the influence of pulsed flow velocity increase and nutrient enrichment. *Sci. Total Environ.* **573**, 594–602 (2016). 27585428 10.1016/j.scitotenv.2016.08.158

[22] Gumiero, B., Mant, J., Hein, T., Elso, J. & Boz, B. Linking the restoration of rivers and riparian zones / wetlands in Europe: sharing knowledge through case studies. *Ecol. Eng.* **56**, 36–50 (2013).

[23] Bruno, M. C., Siviglia, A., Carolli, M. & Maiolini, B. Multiple drift responses of benthic invertebrates to interacting hydropeaking and thermopeaking waves. *Ecohydrology* **6**, 511–522 (2013).

[24] Bondar-Kunze, E., Kasper, V. & Hein, T. Responses of periphyton communities to abrupt changes in water temperature and velocity, and the relevance of morphology: a mesocosm approach. *Sci. Total Environ*. **768**, 145200 (2021). 10.1016/j.scitotenv.2021.145200 33736353

[25] Cushman, R. M. Review of ecological effects of rapidly varying flows downstream from hydroelectric facilities. *North Am. J. Fish. Manag.* **5**, 330–339 (1985).

[26] Deemer, B. R. et al. Experimental reductions in subdaily flow fluctuations increased gross primary productivity for 425 river kilometers downstream. *PNAS Nexus* **1**, 1–12 (2022). 10.1093/pnasnexus/pgac094 PMC9896909 36741441

[27] Kennedy, T. A. et al. Flow management for hydropower extirpates aquatic insects, undermining river food webs. *Bioscience* **66**, 561–575 (2016).

[28] Holzapfel, P., Leitner, P., Habersack, H., Graf, W. & Hauer, C. Evaluation of hydropeaking impacts on the food web in alpine streams based on modelling of fish- and macroinvertebrate habitats. *Sci. Total Environ.* **575**, 1489–1502 (2017). 27789080 10.1016/j.scitotenv.2016.10.016

[29] Abernethy, E. F. et al. Hydropeaking intensity and dam proximity limit aquatic invertebrate diversity in the Colorado River Basin. *Ecosphere* **12**, e03559 (2021).

[30] Vollset, K. W., Skoglund, H., Wiers, T. & Barlaup, B. T. Effects of hydropeaking on the spawning behaviour of Atlantic salmon *Salmo salar* and brown trout *Salmo trutta*. *J. Fish Biol.* **88**, 2236–2250 (2016). 27125209 10.1111/jfb.12985

[31] Bardonnet, A., Gaudin, P. & Persat, H. Microhabitats and diel downstream migration of young grayling (*Thymallus thymallus* L.). *Freshw. Biol.* **26**, 365–376 (1991).

[32] Bardonnet, A., Poncin, P. & Roussel, J.-M. Brown trout fry move inshore at night: a choice of water depth or velocity? *Ecol. Freshw. Fish* **15**, 309–314 (2006).

[33] Bätz, N., Judes, C. & Weber, C. Nervous habitat patches: the effect of hydropeaking on habitat dynamics. *River Res. Appl.* **39**, 349–363 (2023).

[34] Bauersfeld, K. *Stranding of juvenile salmon by flow reductions at Mayfield Dam on the Cowlitz River* (Technical report 38. Washington Department of Fisheries. Olympia, 1978).

[35] Insulaire, F. et al. Characterizing the effects of morphological microstructures and hydropeaks on fish stranding in rivers. *River Res. Appl.* **40**, 834–849 (2024).

[36] Glowa, S. E., Watkinson, D. A., Jardine, T. D. & Enders, E. C. Evaluating the risk of fish stranding due to hydropeaking in a large continental river. *River Res. Appl.* **39**, 444–459 (2023).

[37] Jones, N. E. & Petreman, I. C. Environmental influences on fish migration in a hydropeaking river. *River Res. Appl.* **31**, 1109–1118 (2015).

[38] Bartoň, D. et al. Use of a flow deflector to protect rheophilic fish spawning grounds during hydropeaking. *River Res. Appl.* **39**, 561–569 (2023).

[39] Casas-Mulet, R., Alfredsen, K., Brabrand, A. & Saltveit, S. J. Hydropower operations in groundwater-influenced rivers: Implications for Atlantic salmon, *Salmo salar*, early life stage development and survival. *Fish Manag. Ecol.* **23**, 144–151 (2016).

[40] Schmutz, S. et al. Response of fish communities to hydrological and morphological alterations in hydropeaking rivers of Austria. *River Res. Appl.* **31**, 919–930 (2015).

[41] Bain, M. B., Finn, J. T. & Booke, H. E. Streamflow regulation and fish community structure. *Ecology* **69**, 382–392 (1988).

[42] Judes, C. et al. Consistent but secondary influence of hydropeaking on stream fish assemblages in space and time. *J. Ecohydraulics* **6**, 157–171 (2021).

[43] Alexandre, C. M. et al. Technologies for the study of hydropeaking impacts on fish populations: applications, advantages, outcomes, and future developments. *River Res. Appl.* **39**, 538–553 (2023).

[44] Moreira, M. et al. Ecologically-based criteria for hydropeaking mitigation: a review. *Sci. Total Environ.* **657**, 1508–1522 (2019). 30677917 10.1016/j.scitotenv.2018.12.107

[45] Bradford, M. J. et al. An experimental study of the stranding of juvenile coho salmon and rainbow trout during rapid flow decreases under winter conditions. *North Am. J. Fish. Manag.* **15**, 473–479 (1995).

[46] Bradford, M. J. An experimental study of stranding of juvenile salmonids on gravel bars and in sidechannels during rapid flow decreases. *Regul. Rivers Res. Manag.* **13**, 395–401 (1997).

[47] Halleraker, J. H. et al. Factors influencing stranding of wild juvenile brown trout (*Salmo trutta*) during rapid and frequent flow decreases in an artificial stream. *River Res. Appl.* **19**, 589–603 (2003).

[48] Mameri, D. et al. Cold thermopeaking-induced drift of nase *Chondrostoma nasus* larvae. *Aquat. Sci*. **85**, 56 (2023). 10.1007/s00027-023-00955-x PMC10038962 36987436

[49] Auer, S., Zeiringer, B., Führer, S., Tonolla, D. & Schmutz, S. Effects of river bank heterogeneity and time of day on drift and stranding of juvenile European grayling (*Thymallus thymallus* L.) caused by hydropeaking. *Sci. Total Environ.* **575**, 1515–1521 (2017). 27793351 10.1016/j.scitotenv.2016.10.029

[50] Auer, S., Hayes, D. S., Führer, S., Zeiringer, B. & Schmutz, S. Effects of cold and warm thermopeaking on drift and stranding of juvenile European grayling (*Thymallus thymallus*). *River Res. Appl.* **39**, 401–411 (2023).

[51] Hayes, D. S. et al. The interactive effect of river bank morphology and daytime on downstream displacement and stranding of cyprinid larvae in hydropeaking conditions. *Ecohydrol. Hydrobiol.* **23**, 152–161 (2023).

[52] Führer, S. et al. Stranding of larval nase (*Chondrostoma nasus* L.) depending on bank slope, down-ramping rate and daytime. *Front. Environ. Sci.* **10**, 1–18 (2022).

[53] Führer, S. et al. Variation in hydropeaking-induced stranding of *Barbus barbus* L. and *Chondrostoma nasus* L. larvae: assessing the impact of daytime and down-ramping rates. *Ecohydrology* **17**, e2626 (2024).

[54] Elliott, J. M. *Quantitative Ecology and the Brown Trout* (Oxford University Press, 1994).

[55] Bardonnet, A., Gaudin, P. & Thorpe, J. E. Diel rhythm of emergence and of first displacement downstream in trout (*Salmo trutta*), Atlantic salmon (*S. salar*) and grayling (*Thymallus thymallus*). *J. Fish Biol.* **43**, 755–762 (1993).

[56] Bardonnet, A. & Gaudin, P. Diel pattern of first downstream post-emergence displacement in grayling, *Thymallus thymallus* (L.,1758). *J. Fish Biol.* **37**, 623–627 (1990).

[57] Zitek, A. et al. Fish drift in a Danube sidearm-system: II. Seasonal and diurnal patterns. *J. Fish Biol.* **65**, 1339–1357 (2004).

[58] Zauner, G. & Eberstaller, J. Klassifizierungsschema der österreichischen Flußfischfauna in bezug auf deren Lebensraumansprüche. *Österreichs Fisch.* **52**, 198–205 (1999).

[59] Baras, E. & Nindaba, J. Diel dynamics of habitat use by riverine young-of-the-year *Barbus barbus* and *Chondrostoma nasus* (Cyprinidae). *Arch. Hydrobiol.* **146**, 431–448 (1999).

[60] Auer, S., Fohler, N., Zeiringer, B., Führer, S. & Schmutz, S. *Experimentelle Untersuchungen zur Schwallproblematik - Drift und Stranden von Äschen und Bachforellen während der ersten Lebensstadien* (Bundesamt für Umwelt, 2014).

[61] Chawla, N. V., Bowyer, K. W., Hall, L. O. & Kegelmeyer, W. P. SMOTE: synthetic minority over-sampling technique. *J. Artif. Intell. Res.* **16**, 321–357 (2002).

[62] Breiman, L. Random forests. *Mach. Learn.* **45**, 5–32 (2001).

[63] Breiman, L., Friedman, J. H., Olshen, R. A. & Stone, C. J. *Classification and Regression Trees*. (Wadsworth, 1984).

[64] Štrumbelj, E. & Kononenko, I. Explaining prediction models and individual predictions with feature contributions. *Knowl. Inf. Syst.* **41**, 647–665 (2014).

[65] Lundberg, S. M. et al. From local explanations to global understanding with explainable AI for trees. *Nat. Mach. Intell.* **2**, 56–67 (2020). 32607472 10.1038/s42256-019-0138-9 PMC7326367

[66] Lundberg, S. M. & Lee, S. I. A unified approach to interpreting model predictions. In *Proc. 31st International Conference on Neural Information Processing Systems* 4766–4775 (ACM, 2017).

[67] R Core Team. R: a language and environment for statistical computing (R foundation for statistical computing). Available at: www.R-project.org (2025).

[68] Heggenes, J. et al. Hydropower-driven thermal changes, biological responses and mitigating measures in northern river systems. *River Res. Appl.* **37**, 743–765 (2021).

[69] Donaldson, M. R., Cooke, S. J., Patterson, D. A. & Macdonald, J. S. Cold shock and fish. *J. Fish Biol.* **73**, 1491–1530 (2008).

[70] Galloway, B. J. & Kieffer, J. D. The effects of an acute temperature change on the metabolic recovery from exhaustive exercise in juvenile Atlantic salmon (*Salmo salar*). *Physiol. Biochem. Zool*. **76**, 652–662 (2003). 10.1086/376921 14671713

[71] Larrieu, K. G. & Pasternack, G. B. Automated analysis of lateral river connectivity and fish stranding risks. Part 2: Juvenile Chinook salmon stranding at a river rehabilitation site. *Ecohydrology* **14**, 1–14 (2021).

[72] Hayes, D. S., Hauer, C. & Unfer, G. Fish stranding in relation to river bar morphology and baseflow magnitude: Combining field surveys and hydrodynamic–numerical modelling. *Ecohydrology* **17**, e2616 (2024).

[73] Sobenes, C., Díaz, C. & Sandoval, F. Critical swimming speed at different temperatures for small-bodied freshwater native riverine fish species. *Sci. Rep.* **14**, 1–9 (2024). 39122770 10.1038/s41598-024-69355-x PMC11316079

[74] Bardonnet, A. Use of visual landmarks by young trout (*Salmo trutta*) during their diel downstream post-emergence displacement in experimental channels. *J. Fish Biol.* **43**, 375–384 (1993).

[75] Hayes, D. S. et al. Life stage-specific hydropeaking flow rules. *Sustain**ability* **11**, 1547 (2019).

[76] Bejarano, M. D., Jansson, R. & Nilsson, C. The effects of hydropeaking on riverine plants: a review. *Biol. Rev.* **93**, 658–673 (2018). 28815925 10.1111/brv.12362

[77] Le Coarer, Y., Lizée, M. H., Beche, L. & Logez, M. Horizontal ramping rate framework to quantify hydropeaking stranding risk for fish. *River Res. Appl.* **39**, 478–489 (2023).

[78] Receveur, J. P. et al. Riverine drift communities during larval fish dispersal over multiple recruitment years. *Hydrobiologia* **849**, 4357–4375 (2022).

[79] Schinegger, R., Trautwein, C., Melcher, A. & Schmutz, S. Multiple human pressures and their spatial patterns in European running waters. *Water Environ. J.* **26**, 261–273 (2012). 24899914 10.1111/j.1747-6593.2011.00285.x PMC4038270

[80] Humphries, P. et al. Riverscape recruitment: A conceptual synthesis of drivers of fish recruitment in rivers. *Can. J. Fish Aquat. Sci.* **77**, 213–225 (2020).

[81] Bartoň, D. et al. Effects of hydropeaking on the attached eggs of a rheophilic cyprinid species. *Ecohydrology* **14**, e2280 (2021).

[82] Becker, C. D., Neitzel, D. A. & Fickeisen, D. H. Effects of dewatering on chinook salmon redds: tolerance of four developmental phases to daily dewaterings. *Trans. Am. Fish. Soc*. **111**, 624–637 (1982).

[83] Harnish, R. A., Ham, K. D., Skalski, J. R., Townsend, R. L. & Buchanan, R. A. Factors affecting powerhouse passage of spring migrant smolts at federally operated hydroelectric dams of the Snake and Columbia rivers. *Can. J. Fish Aquat. Sci*. **80**, 1949–1966 (2023).

[84] Schmutz, S., Hayes, S. D. & Auer, S. Hydropeaking strands and displaces larval and juvenile fish across species - dataset and code. https://zenodo.org/uploads/19661575. Accessed 20 April 2026. 10.1038/s43247-026-03580-2 PMC13364723 42454070

