# Supplementary material for: Hydropeaking strands and displaces larval and juvenile fish across species
Source: Commun Earth Environ. 2026 May 7;7(1):592. doi: 10.1038/s43247-026-03580-2 (PMC13364723; doi:10.1038/s43247-026-03580-2)
Supplement: Supplementary file 2 — Supplementary Information [file 43247_2026_3580_MOESM2_ESM.pdf]

Supplementary Information

Hydropeaking strands and displaces larval and juvenile fish across species

Authors: Stefan Schmutz[a]*†, Daniel S. Hayes[a]†, Simon Führer[a], Franz Greimel[a], Bernhard Zeiringer[a], Mathias Jungwirth[a], Stefan Auer[a]†

Affiliations:

[a]BOKU University, Institute of Hydrobiology and Aquatic Ecosystem Management, Department of Ecosystem Management, Climate and Biodiversity, Gregor-Mendel-Straße 33, 1180 Vienna, Austria.

*Corresponding author. Email: stefan.schmutz@boku.ac.at

†These authors contributed equally to this work.

Supplementary Methods

Experimental setup

Even though both flumes are supplied with water from Lake Lunz, our setup avoids pseudoreplication, as the experimental treatments are applied independently, and the shared water source serves only to standardize environmental conditions. Path dependencies and preconditions have been effectively controlled and standardized across both flumes. The flumes are constructed as lateral reversals of each other and prepared identically. Fish are randomly assigned to the flumes to eliminate individual behavioral or physiological biases. To prevent potential hysteresis effects, care was taken to ensure that the water in the mixing tanks was at the same temperature at the start of each trial when both channels were used simultaneously. Flow was continuously monitored in the flumes to detect any hydrological retention effects. Consequently, any observed differences in fish behavior can be confidently attributed to experimental treatments rather than uncontrolled variables.

Flume and mesocosm experiments

Mesocosms experiments were done in four customized mesocosms (2.25 m × 2 m), with two of them each placed in the shore area of both channels. Each mesocosm consists of a steel framed wooden platform covered by an approx. 15 mm thick layer of sand and fine gravel (d_{max} <10 mm; d_{10} = 0.6 mm; d_{50} = 2.2 mm; d_{90} = 6.0 mm), representing a riverbank's water fluctuation area under HPK, exposing fish to the risk of stranding. The lateral slopes of the mesocosms can be adjusted to match the experimental conditions. Along the lower edge of the platform, in the direction of the flow, an aluminum channel (250 × 75 mm) is installed, which simulates the permanently wetted near-shore area of the river. The entire platform is surrounded by a thin rectangular steel frame into which a fine-mesh net (0.75 mm mesh size) is stretched to prevent fish from leaving the mesocosms but permeable enough to ensure adequate flow velocity conditions within the mesocosms.

Due to the use of these nets, our mesocosms represent a closed system in which potential drift behavior could not be adequately studied. The experiments focused on stranding behavior during down-ramping, considering various parameters. Fish found near the downstream net were not classified as stranded, as the net's potential influence on their behavior could not be ruled out (for more details see Führer et al.[53,54]).

At the end of the final phase, observed stranded fish were removed from the substrate, while non-stranded (swimming) fish were removed from the low flow channel. Finally, the discharge was increased to flush out any stranded fish that had not been detected earlier. Each mesocosm was treated as an independent experiment[53,54].

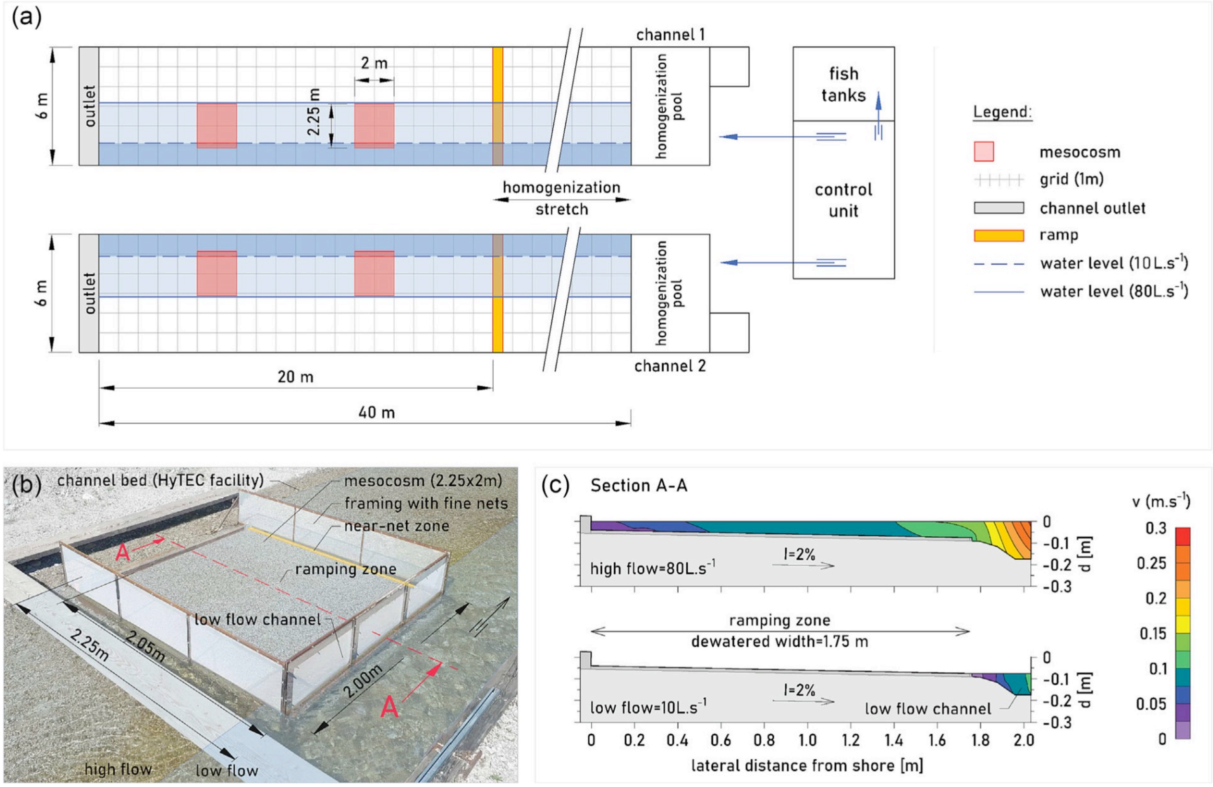

Fig. S1: (a) Overview of the HyTEC facility showing the two experimental channels with details on the mesocosm setup, including water levels at low and high flow. The blue shading indicates the wetted area during the experiments (adapted from Auer et al.[50]). (b) Oblique view of a mesocosm with additional details on features and dimensions. The red line marks the intersection axis for section A–A. (c) Cross-section A–A shows water depth and flow velocity distributions in the mesocosms at high-flow rate of 80 l.s^{-1} (top) and low-flow rate of 10 l.s^{-1} (bottom) (adapted from Führer et al.[54]).

Experiments were conducted in either the left (N=367) or the right channel (N=590). The number of fish tested per experiment ranged from 50–500, depending on fish size and the spatial extent of the experimental setup (full channel or mesocosm). In total, 957 experiments were conducted: 880 with HPK and 77 controls without HPK.

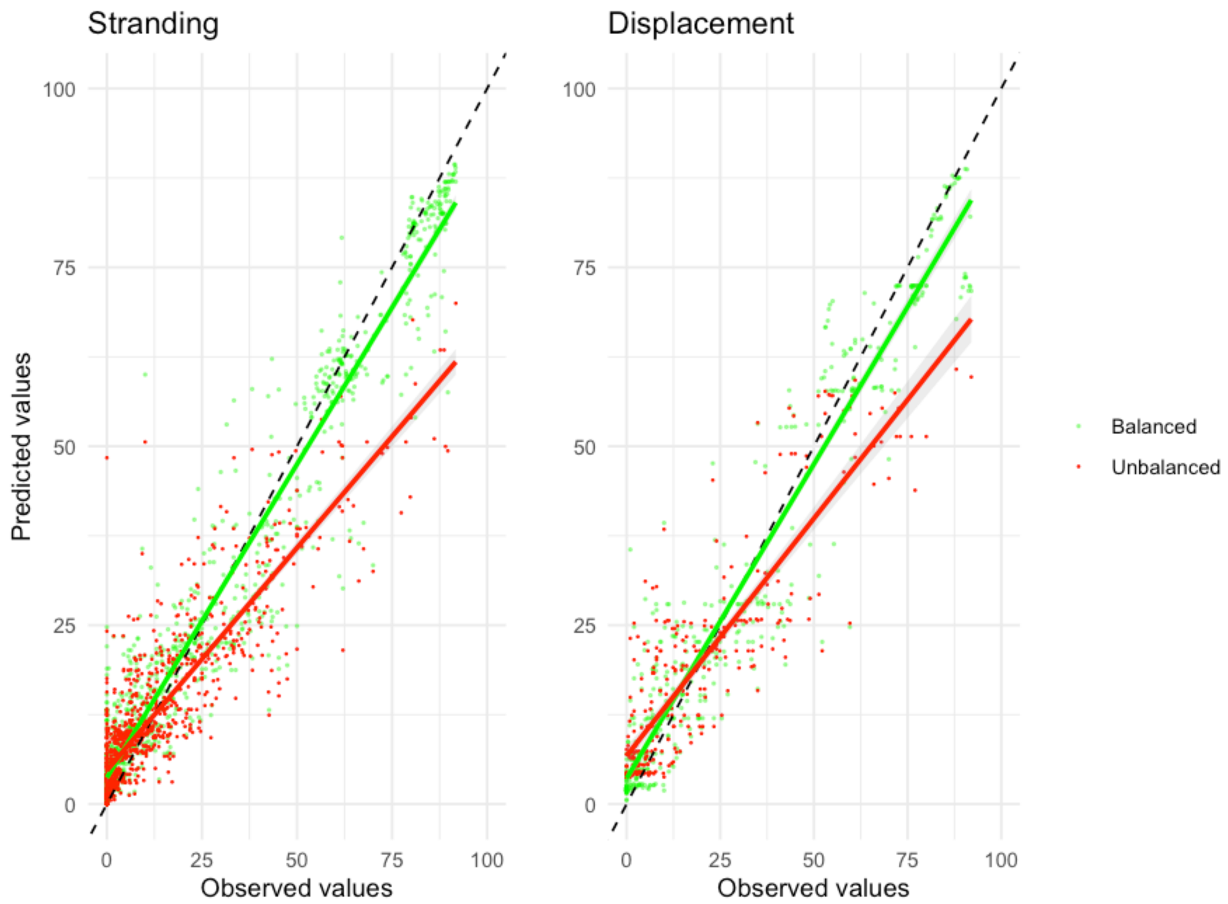

Fig. S2. Comparison of observed versus predicted values for models trained on unbalanced and balanced data. Balancing was performed by subsampling overrepresented cases for stranding and recalculating underrepresented ranges of the target variables (stranding, displacement), resulting in a balanced distribution and improved model fit.

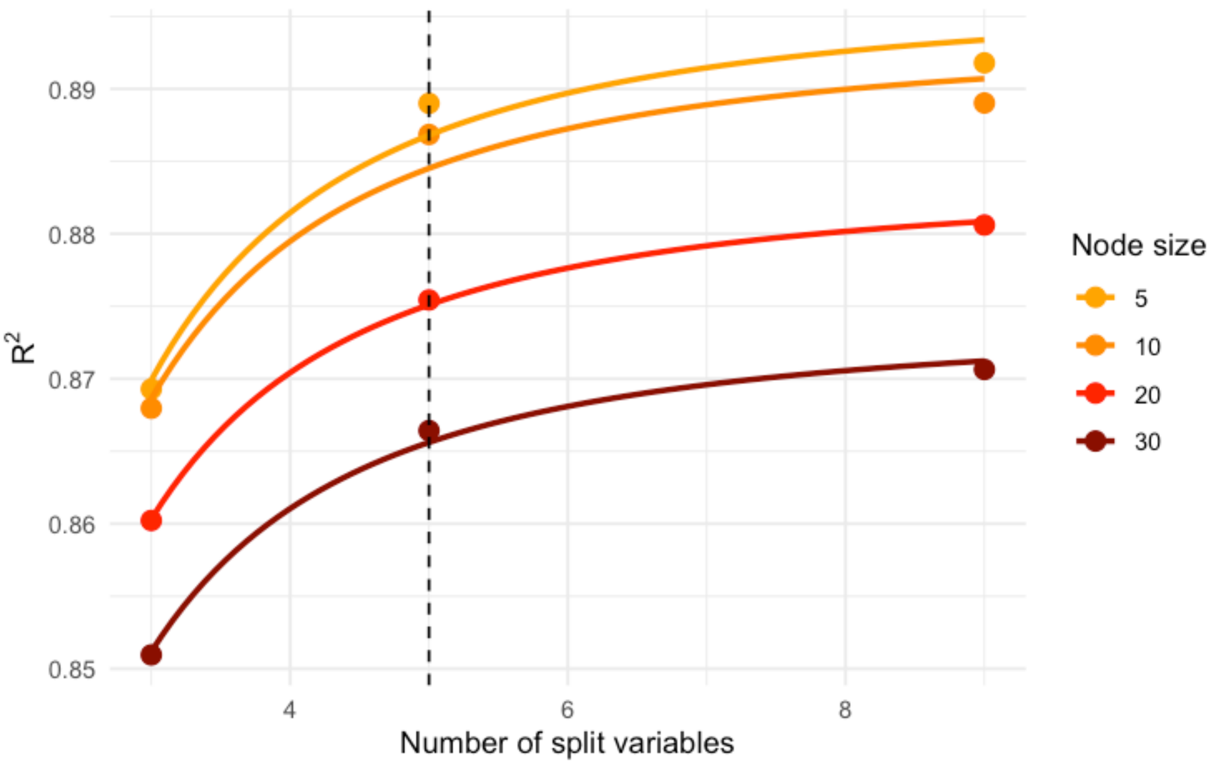

Fig. S3. Optimization of random forest parameters: number of split variables and minimum node sizes. The final models used five variables for splits (dashed line) and a minimum node size of 10 samples.

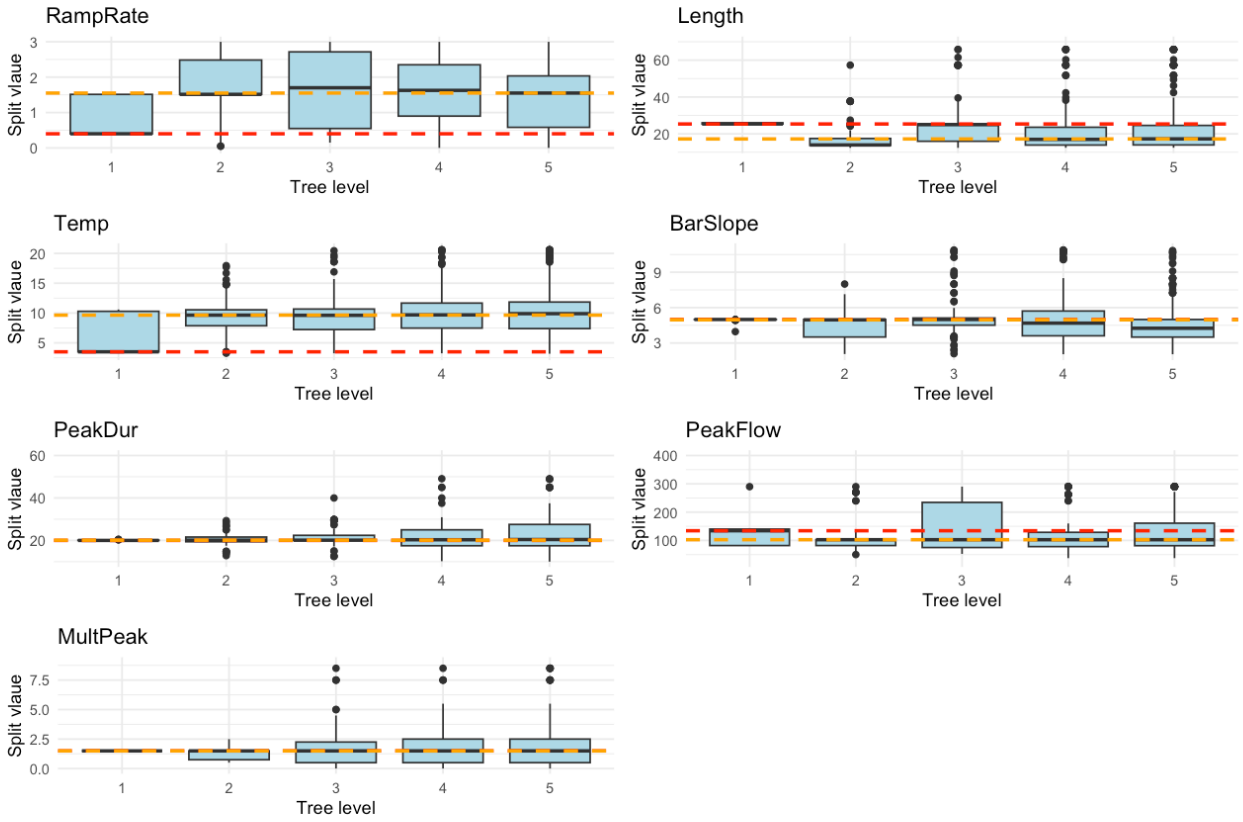

Fig. S4: Split values of numeric variables for the first five tree levels of the random forest model for stranding. Horizontal lines represent median values for the first level (red) and levels 2-5 (orange).

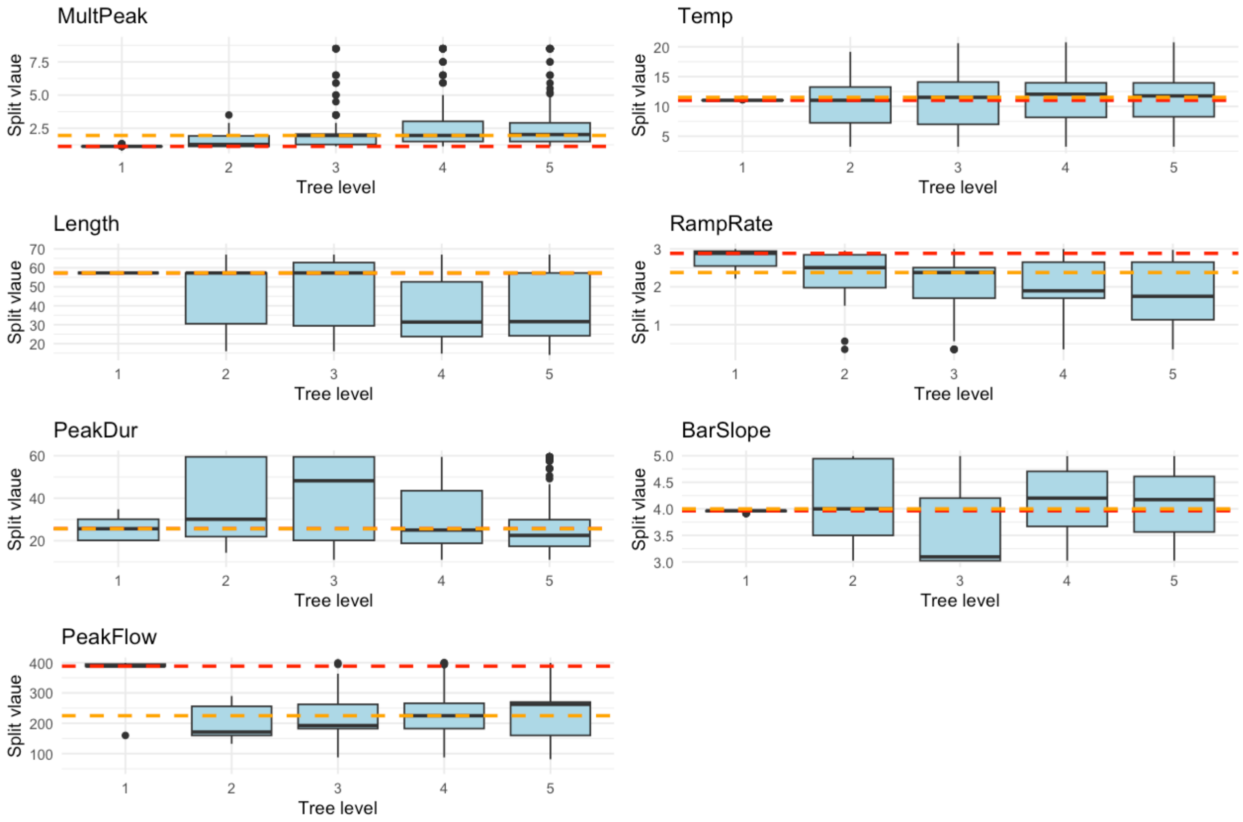

Fig. S5: Split values of numeric variables for the first five tree levels of the random forest model for displacement. Horizontal lines represent median values for the first level (red) and levels 2-5 (orange).

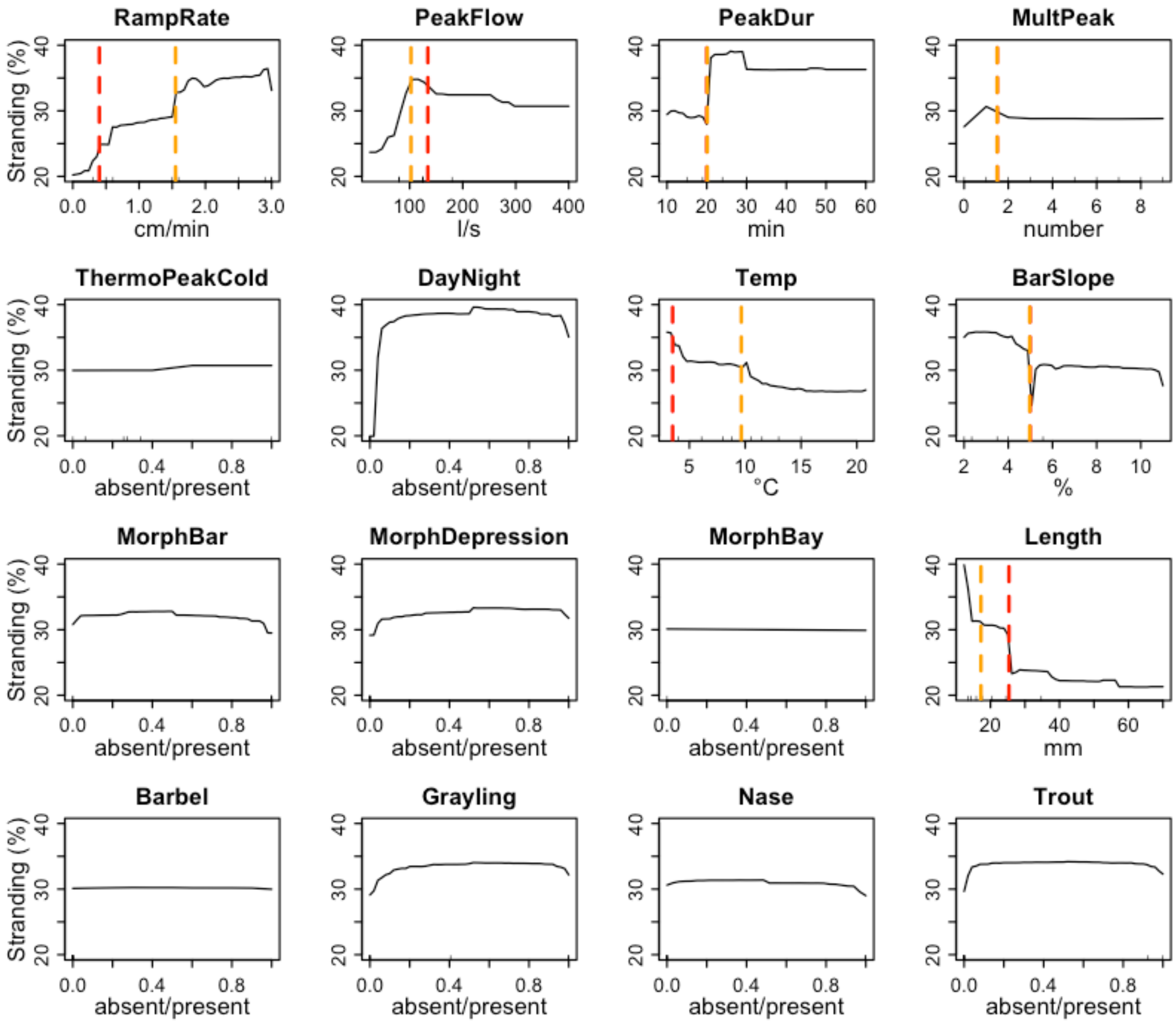

Fig. S6: Partial dependence plots showing the predicted stranding (%) as a function of each predictor. Vertical lines indicate median split values for numeric variables extracted from the random forest models (red: first tree level, orange: levels 2-5; see Table S 5).

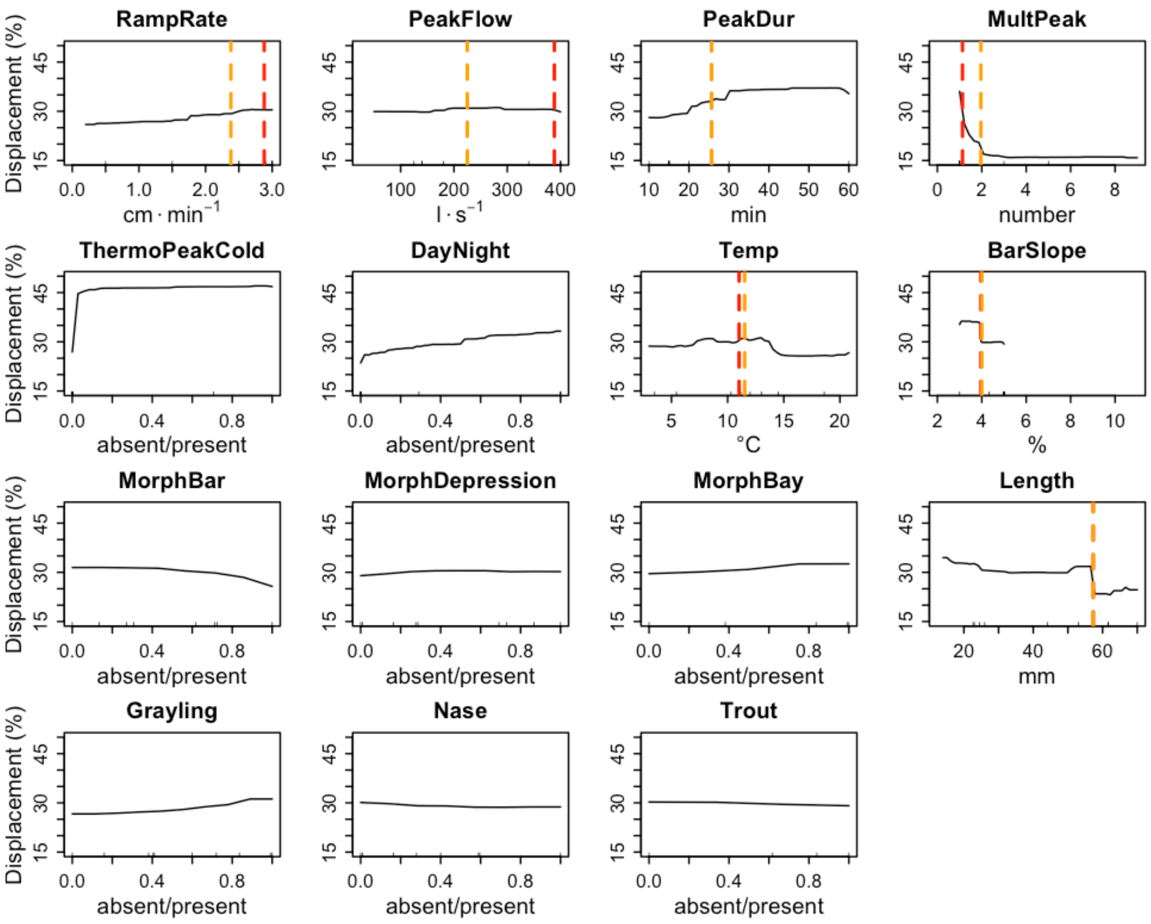

Fig. S7: Partial dependence plots showing the predicted displacement (%) as a function of each predictor. Vertical lines indicate median split values for numeric variables extracted from the random forest models (red: first tree level, orange: levels 2-5; see Table S 5). Note: No displacement experiments were conducted for barbel; therefore, barbel is not shown.

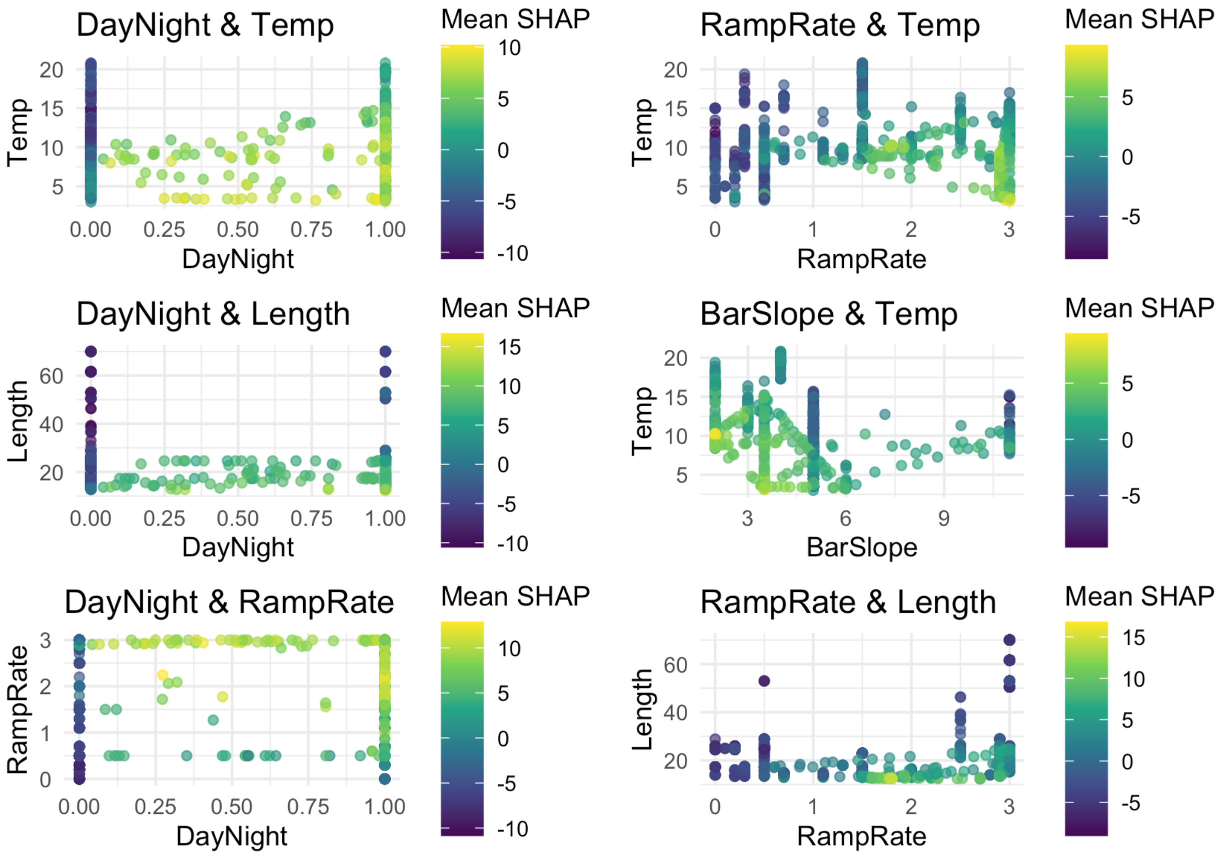

Fig. S8: Interactions between fish stranding (target variable) and the six most important predictors, represented as SHAP values.

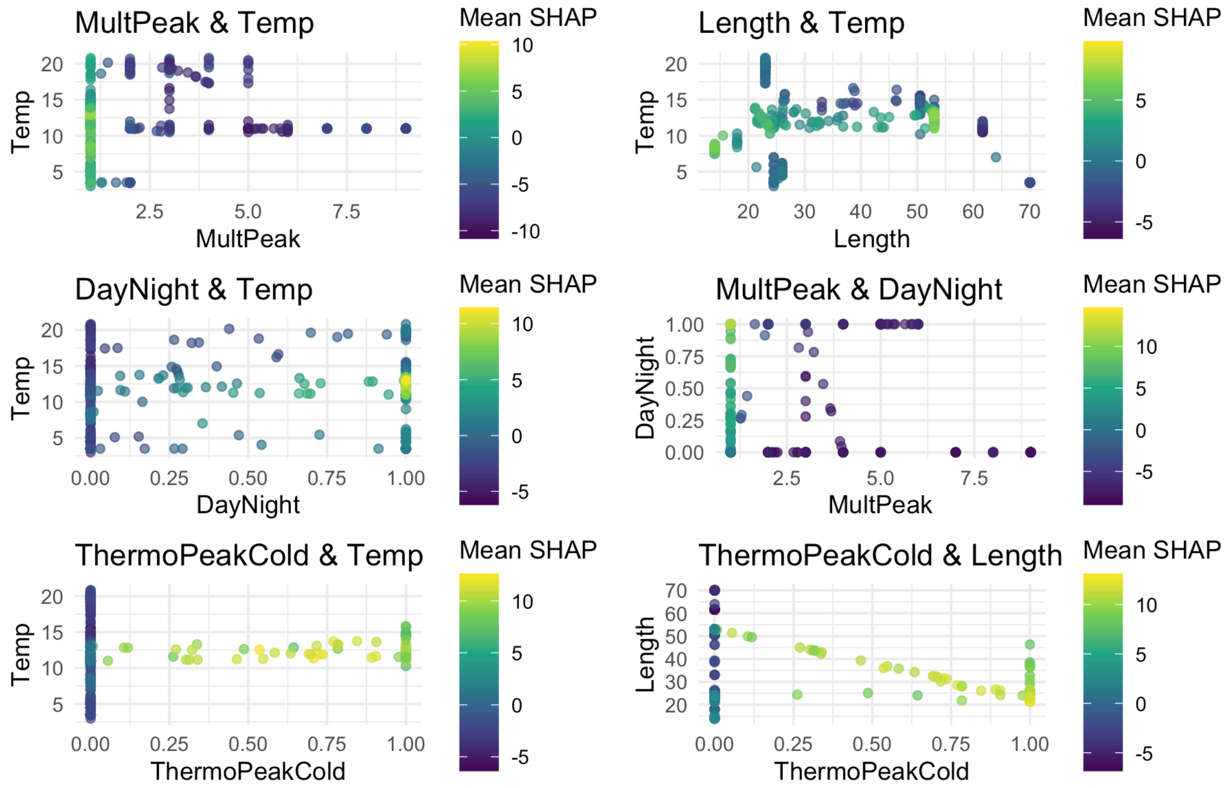

Fig. S9: Interactions between fish displacement (target variable) and the six most important predictors, represented as SHAP values.

Table S 1. Main characteristics and findings of indoor flume stranding experiments.

Source	Species	Fish length (mm)	Peaks[a]	Water temperature (°C)	Ramping rate (cm/h)	Ramping rate (cm.min^{-1})	Day/Night	Added structures[b]	Bar slope (%)	Stranding (%)	Main findings
[45]	Coho salmon	58-88[c]	S	< 4	6	0.10	Day	-	6	~45	High stranding at low temperatures
	Onchorynchus kisutch			< 4	30	0.50	Day	-	6	~72	Higher stranding at higher ramping rates
				< 4	60	1.00	Day	-	6	~40	Higher stranding during the day
				< 4	6	0.10	Day	-	2	~50	Lower stranding at higher slope
				< 4	6	0.10	Night	-	2	~0	Pools increase stranding
				< 4	30	0.50	Day	-	2	~85	Coho salmon has higher stranding than rainbow trout
				< 4	30	0.50	Night	-	2	~10	
				< 4	60	1.00	Day	-	2	~75	
				< 4	60	1.00	Night	-	2	~90	
				< 4	6	0.10	Day	P&C	2	~20	
				< 4	6	0.10	Night	P&C	2	~8	
				< 4	30	0.50	Day	P&C	2	~65	
				< 4	30	0.50	Night	P&C	2	~13	
	Rainbow trout	90[c]	S	< 4	6	0.10	Day	P&C	2	~25	High stranding at low temperatures
	Oncorhynchus mykiss			< 4	6	0.10	Night	P&C	2	~5	Higher stranding at higher ramping rates
				< 4	30	0.50	Day	P&C	2	~42	Higher stranding during the day
				< 4	30	0.50	Night	P&C	2	~10	Pools increase stranding
				< 4	6	0.10	Day	-	2	~6	Rainbow trout has lower stranding than coho salmon
				< 4	6	0.10	Night	-	2	~0	
				< 4	30	0.50	Day	-	2	~27	
				< 4	30	0.50	Night	-	2	~3	
				< 4	60	1.00	Day	-	2	~36	
				< 4	60	1.00	Night	-	2	~6	

Source	Species	Fish length (mm)	Peaks[a]	Water temperature (°C)	Ramping rate (cm/h)	Ramping rate $(cm.min^{-1})$	Day/Night	Added structures[b]	Bar slope (%)	Stranding (%)	Main findings
46	Chinook salmon	40	S	6	6	0.10	Day	-	2	~3	Low stranding, but slight increase at higher ramping rates during the day
	Oncorhynchus tshwytscha			6	6	0.10	Night	-	2	~2	Nearly no stranding at 12°C
				6	30	0.50	Day	-	2	~4	
				6	30	0.50	Night	-	2	~3	
				6	60	1.00	Day	-	2	~10	
				6	60	1.00	Night	-	2	~5	
				12	6	0.10	Day	-	2	~<2	
				12	6	0.10	Night	-	2	~<2	
				12	30	0.50	Day	-	2	~<2	
				12	30	0.50	Night	-	2	~<2	
				12	60	1.00	Day	-	2	~<2	
				12	60	1.00	Night	-	2	~<2	
	Chinook salmon	57	S	10	6	0.10	Day	SC	-	~34[d]	Trapping increases with ramping rate
				10	6	0.10	Night	SC	-	~18[d]	Higher trapping during the night than during the day
	Oncorhynchus tshwytscha			10	30	0.50	Day	SC	-	~32[d]	Higher trapping for chinook than coho salmon at low ramping rates
				10	30	0.50	Night	SC	-	~37[d]	
	Coho salmon	43	S	10	6	0.10	Day	SC	-	~1[d]	Trapping increases with ramping rate
				10	6	0.10	Night	SC	-	~1[d]	Higher trapping during the night than during the day
	Onchorynchus kisutch			10	30	0.50	Day	SC	-	~2[d]	Lower trapping for coho than chinook salmon at low ramping rates
				10	30	0.50	Night	SC	-	~48[d]	
				10	60	1.00	Day	SC	-	~38[d]	
				10	60	1.00	Night	SC	-	~70[d]	
47	Brown trout	56-88[c]	S	6-6.8	60	1.00	Night	-	4.8	22	Higher stranding with increased ramping rate
	Salmo trutta	50-87[c]	S	10-12	60	1.00	Night	-	4.8	<8	Lower temperature increases ramping rate

Source	Species	Fish length (mm)	Peaks[a]	Water temperature (°C)	Ramping rate (cm/h)	Ramping rate (cm.min⁻¹)	Day/Night	Added structures[b]	Bar slope (%)	Stranding (%)	Main findings
			M	10-12	60	1.00	Day	-	4.8	10	Higher stranding at night

[a] Peaks: S=single, M=multiple.
[b] Added structures: P&C=pool and cover, SC=side channel.
[c] Fish were re-used for multiple experiments.
[d] The experiment focused on quantifying trapped fish.

Table S 2. Characteristics of numeric variables (N=957; redundant variables in italics).

Variable	Unit	Code	Mean	SD	Min	25th pctile	75th pctile	Max
Amplitude	*$l.s^{-1}$*	*Ampl*	*123*	*109*	*0*	*70*	*100*	*375*
Bar slope	%	BarSlope	4.5	2	2	3.5	5	11
Base flow	*$l.s^{-1}$*	*BaseFlow*	*19*	*8*	*10*	*10*	*25*	*125*
Displacement[a]	%	Displ	20	21	0	4	30	92
Flow ratio	*ratio*	*FlowRatio*	*7.5*	*4.1*	*1*	*5*	*8*	*16*
Fish length	mm	Length	25	16	12	15	26	70
Multiple peaks	number	MultPeak	1.2	1.2	0	1	1	9
Peak duration	min	PeakDur	22	9	10	20	20	60
Peak flow	$l.s^{-1}$	PeakFlow	142	113	25	80	125	400
Ramping rate lateral	*$cm.min^{-1}$*	*RampLat*	*41*	*28*	*0*	*10*	*60*	*100*
Ramping rate vertical	$cm.min^{-1}$	RampRate	1.6	1.1	0	0.5	3	3
Stranding	%	Stran	12	17	0	0.59	17	92
Temperature	°C	Temp	10	4.2	3	7.9	13	21

[a]Displacement data are only available for a subset of the data (N=287).

Table S 3. Description, number and percentage of tested categorical variables.

**Variable (Code)**	**Description**	**N**	**%**
DayNight		957	
... Day	1 h after sunrise - 1 h before sunset	611	64%
... Night	1 h after sunset - 1 h before sunset	346	36%
Morphology		957	
... Bay (MorphBay)	Fully wetted during base and peak flow	22	2%
... Depression (MorphDepression)	Postponed dewatering in depressions	276	29%
... Gravel bar (MorphBar)	Regressive dewatering	659	69%
Species	Length: mean (min-max)	957	
... Barbel	14.4 mm (13.4-14.9)	63	7%
... European Grayling	34.2 mm (13.5-70.0)	364	38%
... Nase	16.8 mm (12.4-23.0)	344	36%
... Brown trout	25.8 mm (24.5-28.9)	186	19%
Thermopeaking		957	
... cold (ThermoPeakCold)	~3°C decrease	15	2%
... hot (ThermoPeakHot)	~3°C increase	16	2%

Variable (Code)	Description	N	%
... no	No change	926	96%

Table S 4. Performance of models trained on balanced and unbalanced datasets for stranding and displacement. Mean values across 500 trees for mean squared error (MSE) and R^2 (HPK=hydropeaking).

Model	Data	Variables	N	MSE	R^2
Stranding	balanced	HPK	956	275.8	0.697
	balanced	all	956	109.5	0.880
	unbalanced	all	957	112.8	0.602
Displacement	balanced	HPK	481	303.5	0.657
	balanced	all	481	105.7	0.880
	unbalanced	all	287	150.1	0.643

Table S 5. Split values for the random forest models of stranding and displacement for the first level and levels 2-5. See also Figs. S4-S5.

Model	Variable	Unit	Median (Level 1)	Median (Levels 2-5)
Stranding	RampRate	$cm.min^{-1}$	0.40	1.59
Stranding	Length	mm	25.36	17.03
Stranding	Temp	°C	3.50	9.67
Stranding	BarSlope	%	4.99	4.82
Stranding	PeakFlow	$l.s^{-1}$	134.22	102.50
Stranding	PeakDur	min	20.01	20.22
Stranding	MultPeak	number	1.50	1.50
Displacement	MultPeak	number	1.13	1.96
Displacement	Temp	°C	11.02	11.52
Displacement	Length	mm	57.30	57.30
Displacement	RampRate	$cm.min^{-1}$	2.88	2.38
Displacement	PeakDur	min	25.65	25.65
Displacement	BarSlope	%	3.96	4.00
Displacement	PeakFlow	$l.s^{-1}$	388.40	225.00

Table S 6. Correlation between model residuals and experimental settings.

Response	Variable	N	Correlation	t_value	p_value_adj
Stranding	Channel side	957	0.029	0.901	1.0000
Stranding	Full channel/Mesocosm	957	0.069	2.131	0.1000
Stranding	Stocking density	957	0.028	0.865	1.0000
Displacement	Channel side	287	-0.061	-1.032	0.6055
Displacement	Stocking density	287	0.122	2.072	0.0783
