# Supplementary material for: Hydropeaking strands and displaces larval and juvenile fish across species
Source: Commun Earth Environ. 2026 May 7;7(1):592. doi: 10.1038/s43247-026-03580-2 (PMC13364723; doi:10.1038/s43247-026-03580-2)
Supplement: Supplementary file 1 — Transparent Peer Review file [file 43247_2026_3580_MOESM1_ESM.pdf]

nature portfolio

Peer Review File

Hydropeaking strands and displaces larval and juvenile fish across species

Corresponding Author: Professor Stefan Schmutz

This file contains all editorial decision letters in order by version, followed by all author rebuttals in order by version.

Version 0:

Decision Letter:

** Please ensure you delete the link to your author home page in this e-mail if you wish to forward it to your coauthors **

Dear Professor Schmutz,

Your manuscript titled "Stranded and displaced fish: The ecological toll of hydropeaking" has now been seen by 3 reviewers, and we include their comments at the end of this message. They find your work of interest, but some important points are raised. We are interested in the possibility of publishing your study in Communications Earth & Environment, but would like to consider your responses to these concerns and assess a revised manuscript before we make a final decision on publication.

We therefore invite you to revise and resubmit your manuscript, along with a point-by-point response that takes into account the points raised.

Specifically, please carefully explain your methodology and expand on the interpretation of the figures (including in Supplementary Information), in light of reviewer 3's queries and comments.

An updated and completed version of our Reporting Summary must be uploaded with the revised manuscript.
You can download the form here:
https://www.nature.com/documents/nr-reporting-summary.pdf

Please highlight all changes in the manuscript text file.

Please submit your point-by-point responses as a separate file, distinct from your cover letter where you can add responses to the Editors' comments that you do not want to be made available to the reviewers. Word files are preferred. We recommend that any figures, tables or graphs that are included in the response to reviewers are also included in the main article or Supplementary Information.

We are committed to providing a fair and constructive peer-review process. Please don't hesitate to contact us if you wish to discuss the revision in more detail.

Please use the following link to submit your revised manuscript, point-by-point response to the referees' comments (which should be in a separate document to any cover letter), a tracked-changes version of the manuscript (as a PDF file) and the completed checklist:

Link Redacted
** This url links to your confidential home page and associated information about manuscripts you may have submitted or be reviewing for us. If you wish to forward this email to co-authors, please delete the link to your homepage first **

We hope to receive your revised paper within six weeks; please let us know if you aren't able to submit it within this time so that we can discuss how best to proceed. If we don't hear from you, and the revision process takes significantly longer, we may close your file. In this event, we will still be happy to reconsider your paper at a later date, as long as nothing similar has been accepted for publication at Communications Earth & Environment or published elsewhere in the meantime.

Please do not hesitate to contact us if you have any questions or would like to discuss these revisions further. We look forward to seeing the revised manuscript and thank you for the opportunity to review your work.

Best regards,

Heike Langenberg, PhD
Chief Editor
Communications Earth & Environment
Communications Sustainability

On Bluesky:
@commsearth.nature.com
@commssustain.nature.com

EDITORIAL POLICIES AND FORMATTING

We ask that you ensure your manuscript complies with our editorial policies. Please ensure that the following formatting requirements are met, and any checklist relevant to your research is completed and uploaded as a Related Manuscript file type with the revised article.

Furthermore, please align your manuscript with our format requirements, which are summarized on the following checklist: Communications Earth & Environment formatting checklist

and also in our style and formatting guide Communications Earth & Environment formatting guide .

*** DATA: Communications Earth & Environment endorses the principles of the Enabling FAIR data project (http://www.copdess.org/enabling-fair-data-project/). We ask authors to make the data that support their conclusions available in permanent, publically accessible data repositories. (Please contact the editor if you are unable to make your data available).

All Communications Earth & Environment manuscripts must include a section titled "Data Availability" at the end of the Methods section or main text (if no Methods). More information on this policy, is available at http://www.nature.com/authors/policies/data/data-availability-statements-data-citations.pdf.

In particular, the Data availability statement should include:
- Unique identifiers (such as DOIs and hyperlinks for datasets in public repositories)
- Accession codes where appropriate
- If applicable, a statement regarding data available with restrictions
- If a dataset has a Digital Object Identifier (DOI) as its unique identifier, we strongly encourage including this in the Reference list and citing the dataset in the Data Availability Statement.

DATA SOURCES: All new data associated with the paper should be placed in a persistent repository where they can be freely and enduringly accessed. We recommend submitting the data to discipline-specific, community-recognized repositories, where possible and a list of recommended repositories is provided at http://www.nature.com/sdata/policies/repositories.

If a community resource is unavailable, data can be submitted to generalist repositories such as figshare or Dryad Digital Repository. Please provide a unique identifier for the data (for example a DOI or a permanent URL) in the data availability statement, if possible. If the repository does not provide identifiers, we encourage authors to supply the search terms that will return the data. For data that have been obtained from publically available sources, please provide a URL and the specific data product name in the data availability statement. Data with a DOI should be further cited in the methods reference section.

Please refer to our data policies at http://www.nature.com/authors/policies/availability.html.

REVIEWER COMMENTS:

Reviewer #1 (Remarks to the Author):

The study described in this manuscript investigates the ecological impacts of hydropeaking (HPK)—rapid, intermittent flow fluctuations caused by hydropower operations—on juvenile fish. Using a near-natural experimental facility (HyTEC),

researchers conducted ~1,000 trials with >120,000 fish from four species (brown trout, European grayling, nase, and barbel) to understand how HPK intensity, environmental conditions, and biological factors influence stranding and downstream displacement.

Key results of the manuscript include:
• Hydropeaking intensity (ramping rate, peak duration, peak flow, number of peaks) is the dominant driver of fish stranding and displacement.
• Cold thermopeaking (rapid temperature drops during HPK) amplifies displacement.
• Nighttime conditions, small fish size, and low water temperatures significantly increase stranding risk.
• Behavioral adaptation: Displacement decreases after the first peak, suggesting learning, but stranding remains high.
• Species differences are minor; fish size matters more than species.
• Gravel bar slope and morphology influence outcomes—flat slopes increase stranding.

The study emphasizes that mitigation strategies should consider multiple factors beyond ramping rate, including time of day, fish size, and thermal changes.

Validity
I do not see any flaws that should prohibit the publication of this manuscript.

Originality and significance
Several of the results appear to be original to this manuscript, including the effects of environmental conditions (e.g., water temperature and diel period) on stranding and displacement. The paper also provides quantitative thresholds for ramping rate, temperature, and fish size that can inform mitigation strategies. These results are of immediate relevance to fish ecologists, managers, dam owner/operators, and policy makers, and they extend to hydropower engineering, environmental regulation, and renewable energy planning, making the study significant across multiple disciplines.

Data & methodology
The approach is methodologically sound and well-aligned with the study objectives. The use of a near-natural experimental facility (HyTEC) with controlled hydropeaking scenarios provides ecological realism while maintaining experimental control. The large dataset—nearly 1,000 trials involving >120,000 fish—offers strong statistical power and species coverage across larval and juvenile stages. The experimental design effectively isolates hydropeaking effects by controlling confounding variables (e.g., temperature, morphology).
The data are extensive, covering multiple species, size classes, diel conditions, and hydropeaking intensities. Random forest modeling and SHAP analysis are appropriate for capturing non-linear relationships and interactions among multiple predictors. Threshold identification is supported by robust variance partitioning and cross-validation. Extended data tables and figures provide detailed variable importance, partial plots, and interaction analyses.
The methodology is described in detail, including experimental phases (stocking, acclimation, up-/down-ramping), facility specifications (channel dimensions, flow control, temperature mixing), and statistical procedures (SMOTE balancing, random forest parameters, SHAP interpretation). Extended Data and supplementary materials include threshold values, model performance metrics, and interaction plots. Data and code availability via Zenodo DOI ensures reproducibility.
Figures and tables are clear, with appropriate legends and references to Extended Data. Partial dependence plots and SHAP visualizations enhance interpretability. Some complexity in Extended Data may require careful reading, but overall presentation is strong.

Appropriate use of statistics and treatment of uncertainties
The study uses random forest models with cross-validation, variable importance metrics, and SHAP analysis to interpret predictor contributions. These methods are appropriate for handling complex, non-linear relationships and interactions in large datasets. The approach of using mean split values from tree levels and partial dependence plots is valid for identifying ecological thresholds. The application of SMOTE to address class imbalance is correctly implemented and described.
The main figures (e.g., variable importance, partial plots) do not display traditional error bars because they represent model-derived relationships rather than raw experimental means. Extended Data figures include partial dependence plots and SHAP values, which inherently convey uncertainty through model variance rather than confidence intervals. Probability values (p-values) are reported for correlation analyses (e.g., $r = 0.623$, $p < 0.001$), and these appear accurate and appropriately interpreted.

Conclusions
Overall, the conclusions and data interpretation appear robust and well-supported by the experimental design and statistical analysis. The large sample size (>120,000 fish across nearly 1,000 trials) and near-natural experimental conditions provide strong ecological realism and statistical power. The use of advanced modeling techniques (random forests, SHAP) is appropriate for capturing complex, non-linear relationships and interactions among hydropeaking intensity, environmental factors, and biological traits.

Suggested improvements
I wonder whether the reduction in displacement after the first peak observed in treatments with multiple hydropeaking events, which the authors attributed to learning, might instead be caused by a change in the size distribution of remaining fish. The study acknowledges that stranding and displacement probabilities are strongly size-dependent—smaller fish (<17 mm) have roughly twice the stranding risk compared to larger individuals (>25 mm), and displacement also decreases slightly with increasing size. In treatments with multiple hydropeaking events, fish that survive the first peak are likely to be larger on average than those initially stocked, simply because smaller individuals are more prone to stranding or drifting out

early. This could bias subsequent observations by artificially lowering stranding and drift rates in later peaks, making it appear that fish are “adapting” behaviorally when part of the effect may be due to a shift in size distribution. The authors interpret reduced displacement after the first peak as a learning effect, but they do not explicitly control for or quantify changes in size composition across consecutive peaks. Therefore, some of the observed adaptation could indeed be confounded by size-selective survival. This deserves either some level of discussion in the manuscript or additional data of how the size distribution changes over multiple peaking events (if available).

References
The manuscript generally references previous literature thoroughly and appropriately. It cites foundational work on hydropeaking impacts (e.g., Bradford et al., 1995; Halleraker et al., 2003), temperature effects (Donaldson et al., 2008), diel behavior (Bardonnet et al., 1991, 2006), and recent advances in ecohydraulics and thermopeaking (Auer et al., 2023; Hayes et al., 2019). The reference list is extensive and includes both classic studies and recent systematic reviews, which strengthens the context. Interestingly, a recent paper by Harnish et al. (2023) found that many of the same biological (i.e., fish size) and environmental factors (i.e., flow, temperature, diel period) that affect stranding and displacement also affect the probability of powerhouse entrainment. Harnish et al. (2023) attributed the observed effects to changes in the swimming ability and activity level of fish under varying conditions. The authors may consider reviewing that paper.

Harnish, R.A., K.D. Ham, J.R. Skalski, R.L. Townsend, and R.A. Buchanan. 2023. Factors affecting powerhouse passage of spring migrant smolts at federally operated hydroelectric dams of the Snake and Columbia rivers. Canadian Journal of Fisheries and Aquatic Sciences 80(12):1949-1966.

Clarify and context
The abstract is generally clear and well-structured, effectively summarizing the research problem, methodology, key findings, and implications. It highlights the novelty and practical relevance of the study. The introduction is comprehensive and appropriate, providing strong context, a thorough literature review, and clearly stated hypotheses. It logically progresses from identifying knowledge gaps to explaining the study design. The conclusions appropriately synthesize results and link them to management implications, stressing the need for multi-factor mitigation strategies. While practical recommendations are well articulated, the interpretation of behavioral adaptation (learning effect) is presented with confidence and would benefit from a cautionary note acknowledging potential confounding factors such as size-selective survival. Overall, these sections are well-developed and suitable for the intended audience.

Inflammatory material
The manuscript does not contain language that is inappropriate or potentially libelous.

Reviewer #2 (Remarks to the Author):

The manuscript addresses an important and timely topic: the effects of hydropeaking on fish, with a particular focus on stranding/displacement processes. A major strength of the study is the exceptional experimental dataset, collected over an impressive 10-year period with extensive replication in a truly unique experimental facility. The work clearly builds on a set of prior publications derived from the same experimental system; these studies appear essential for context and interpretation and are appropriately cited.

Overall, the manuscript is well written and I consider it suitable for publication after minor revision, mainly to improve structure, flow, and the breadth of interpretation. In its current (journal-driven) format, a large amount of key information (including all tables) appears in the Extended Data. Also for me non-traditional structure (apparently following journal format)where results are combined with a discussion results in too brief discussion, while broader interpretation remains rather limited. I recommend strengthening the discussion and embedding the findings in a wider ecological and life-history context. In particular, interspecific differences could be more clearly highlighted and interpreted, and the section headings and logical progression of the manuscript could be reconsidered to improve readability and narrative continuity.

A key point that should be clarified already at the outset is the scope of the study with respect to fish size and life stage. The work focuses on young/small fish (up to ~70 mm), which is well justified later in the text, but this should be stated explicitly in the title and/or abstract because it strongly conditions interpretation and generality. Related to this, while the manuscript argues convincingly that early post-hatch stages experience a major bottleneck and are therefore especially relevant, it would be valuable to acknowledge more explicitly that hydropeaking-related disturbance of spawning may also lead to loss of eggs through displacement and subsequent mortality (e.g., drying or detachment from the substrate). These pre-hatch processes can also translate into substantial losses of potential recruits and deserve explicit mention to provide a more balanced life-stage framing.

The introduction and presentation of the experimental facility could also be streamlined. The HyTEC facility is described well in detail in the Methods, but, a brief introduction of how it looks and work could be used early on, supported by an improved Figure 1: in addition to illustrating key river concepts and impacts, the figure could include a simple schematic of how those processes are represented in the experimental system (complementing the figure 1 in Extended Data). In terms of terminology, “ramping rate” does not need quotation marks; it should simply be defined once and then used consistently. Where “thermopeaking” is mentioned, the expected impacts and relevance could be introduced more clearly. The final sentence of the first section of the main text appears to require a citation. Species naming should also be consistent; grayling should be specified allways as European grayling.

Several figure-related improvements would enhance accessibility. Figure 2 would benefit from using full variable names rather than codes (even if codes are defined in Extended Data), and the ordering could be adjusted so that the most important variables appear at the top for easier interpretation. Peak flow should be reported in unit $m^3.s^{-1}$ (btw. why PeakFlow shows no importance for displacement in fg 2?) In the text, wording “Other HPK variables…” could be revised to “Besides ramping rate…” (or similar) to improve clarity. In Figure 3, the visual hierarchy of labels could be improved, as “Hydropeaking variables” and “Stranding” currently appear at the same conceptual level (likewise for “other variables”)

The Conclusions are clearly written and communicate the main outcomes effectively. However, given that the manuscript introduces five hypotheses at the beginning, the Conclusions (and ideally the abstract at a high level) should explicitly state whether each hypothesis is supported or not, so that readers can readily extract the answers to the stated research questions. Finally, while the Methods are generally detailed and strong, they could be expanded with a concise description of the selected experimental species (key ecological traits, life-history considerations, and rationale for selection), which would also help interpret the interspecific patterns.

In summary, this is a strong and valuable contribution based on a rare long-term, highly replicated experimental dataset. With minor revisions focused on clarifying scope (young/small fish), improving structure and narrative flow, strengthening discussion (especially regarding interspecific differences and broader context), explicitly answering the stated hypotheses, and making a few targeted improvements to figures and terminology, the manuscript should be well positioned for publication.

Reviewer #3 (Remarks to the Author):

Thanks for the opportunity to review this manuscript. The authors do a great job of explaining the relevance of the work and have an extremely large dataset collected in a purpose-built facility. The writing is very clear and concise until the methods section. I will acknowledge that I know very little about random forests, beyond it being a decision tree and machine learning method. I will defer to other reviewers on the specifics. However, I noticed some pretty glaring problems, either from incorrect use of the methods, lack of explanation, or lack of care/polish in the final product.
First, I had a good idea of what the response variable was, but I had to look at the figure axis in one of the supplemental submissions to confirm you were using a percentage as the response. How was this determined? What was the experimental unit? I assume for each test, you recorded a single % for stranded/displaced, and this was the response. What about for the mesocosm experiments? Did you treat the 4 mesocosms as independent within a single test flow? How did you incorporate fish lengths, since those go to a single fish? Did you take the average length for a test flow? The descriptions of the data used in the study are lacking, even in the supplemental materials. Many of the figures do not have adequate captions.
The most concerning aspect is the figures themselves. There are several binary, categorical predictors (present/absent). The figure show the model estimating values in between, which don’t exist. If there was a straight line between the means, sure, I can see what’s going on, but many have non-linear relationships connecting absent to present. I don’t know what this means, I don’t know if this is a nuance of the model, I don’t know if you’ve mis-specified the model. What it does mean is that nobody looked at the figures critically. Look at extended figure 7, barbel: it’s a blank graph. What are we supposed to be learning from these? The lack of care in the presentation of the data does not give confidence for the amount of care put into the analysis.
Finally, I’m not sure what readers are supposed to learn from this. Ramping rate, as suspected, is the most important aspect of stranding and displacement. However, there’s other factors, none of which are surprising at all. How does this get used? What are the recommendations to minimize the negative effects of hydropower? Using these results and applying them to a field study to show how the experimental study improves management of fish subjected to hydropeaking flow regimes would be widely applicable and useful for conservation. Here, you’re just highlighting other factors that lead to stranding, none of which are surprising.

** Visit Nature Portfolio's author and referees' website at www.nature.com/authors for information about policies, services and author benefits**

Communications Earth & Environment is committed to improving transparency in authorship. As part of our efforts in this direction, we are now requesting that all authors identified as ‘corresponding author’ create and link their Open Researcher and Contributor Identifier (ORCID) with their account on the Manuscript Tracking System prior to acceptance. ORCID helps the scientific community achieve unambiguous attribution of all scholarly contributions. You can create and link your ORCID from the home page of the Manuscript Tracking System by clicking on ‘Modify my Springer Nature account’ and following the instructions in the link below. Please also inform all co-authors that they can add their ORCIDs to their accounts and that they must do so prior to acceptance.
https://www.springernature.com/gp/researchers/orcid/orcid-for-nature-research

For more information please visit http://www.springernature.com/orcid

If you experience problems in linking your ORCID, please contact the Platform Support Helpdesk.

Version 1:

Decision Letter:

<*** REMEMBER TO ATTACH REVISIONS CHECKLIST (WORD)***>

** Please ensure you delete the link to your author home page in this e-mail if you wish to forward it to your coauthors **

Dear Dr Schmutz,

Your manuscript titled "Stranded and displaced fish: The ecological toll of hydropeaking" has now been seen by our reviewers, whose comments appear below. In light of their advice we are delighted to say that we are happy, in principle, to publish a suitably revised version in Communications Earth & Environment.

At the same time we ask that you edit your manuscript to comply with our format requirements and to maximise the accessibility and therefore the impact of your work.

Additionally, the manuscript must include an ethics statement identifying the institutional and/or licensing committee approving the experiments, including any relevant details. You can see details on our policies here: https://www.nature.com/commsenv/editorial-policies/ethics-and-biosecurity#studies-involving-animals-and-human-research-participants.

EDITORIAL REQUESTS:

Please review our specific editorial comments and requests regarding your manuscript in the attached "Editorial Requests Table".

*****Please take care to match our formatting and policy requirements. We will check revised manuscript and return manuscripts that do not comply. Such requests will lead to delays. *****

Please outline your response to each request in the right hand column. Please upload the completed table with your manuscript files as a Related Manuscript file.

If you have any questions or concerns about any of our requests, please do not hesitate to contact me.

SUBMISSION INFORMATION:

In order to accept your paper, we require the files listed at the end of the Editorial Requests Table; the list of required files is also available at https://www.nature.com/documents/commsj-file-checklist.pdf .

OPEN ACCESS:

Communications Earth & Environment is a fully open access journal. Articles are made freely accessible on publication. For further information about article processing charges, open access funding, and advice and support from Nature Portfolio, please visit https://www.nature.com/commsenv/open-access

At acceptance, you will be provided with instructions for completing the open access licence agreement on behalf of all authors. This grants us the necessary permissions to publish your paper. Additionally, you will be asked to declare that all required third party permissions have been obtained, and to provide billing information in order to pay the article-processing charge (APC).

Please use the following link to submit the above items:
Link Redacted
** This url links to your confidential home page and associated information about manuscripts you may have submitted or be reviewing for us. If you wish to forward this email to co-authors, please delete the link to your homepage first **

We hope to hear from you within two weeks; please let us know if you need more time.

Best regards,

Marisa McDonald, PhD

Associate Editor, Communications Earth & Environment
Consulting Editor, Communications Sustainability

On Bluesky:
@commsearth.nature.com
@commssustain.nature.com

REVIEWERS' COMMENTS:

Reviewer #1 (Remarks to the Author):

The authors have adequately addressed my comments and concerns from the initial submission, and I have no further comments. Nice work!

Reviewer #2 (Remarks to the Author):

I have carefully reviewed the revised version of the manuscript and am pleased to say that the authors have addressed the concerns raised in the previous round of review in a thorough and satisfactory manner. The revisions are thoughtful, well executed, and clearly improve the overall quality, clarity, and rigor of the work.

In my view, the authors have responded appropriately to the reviewers' comments and have made the necessary changes with due care and attention. The revised manuscript is now substantially strengthened, and I am very satisfied with its current form.

Overall, I believe the manuscript is suitable for publication, and I would be happy to recommend its acceptance.

** Visit Nature Portfolio's author and referees' website at www.nature.com/authors for information about policies, services and author benefits**

Open Access This Peer Review File is licensed under a Creative Commons Attribution 4.0 International License, which permits use, sharing, adaptation, distribution and reproduction in any medium or format, as long as you give appropriate credit to the original author(s) and the source, provide a link to the Creative Commons license, and indicate if changes were made.
In cases where reviewers are anonymous, credit should be given to 'Anonymous Referee' and the source.
The images or other third party material in this Peer Review File are included in the article's Creative Commons license,

unless indicated otherwise in a credit line to the material. If material is not included in the article's Creative Commons license and your intended use is not permitted by statutory regulation or exceeds the permitted use, you will need to obtain permission directly from the copyright holder.
To view a copy of this license, visit https://creativecommons.org/licenses/by/4.0/

Dear reviewers,

thank you for your time and effort in revising the paper as well as for your overall positive review. Your feedback has helped us to resolve any uncertainties in the article and to better present the results and lessons learned. We believe that we have been able to significantly improve the paper based on your comments.

Below you will find a point-to-point response to the suggestions for improvement. Reviewer comments are in black font, and our responses are in blue font. A reference list of papers cited in the rebuttal can be found at the end of this document.

Yours sincerely,

Stefan Schmutz,

on behalf of the author team

Reviewer #1 (Remarks to the Author):

The study described in this manuscript investigates the ecological impacts of hydropeaking (HPK)—rapid, intermittent flow fluctuations caused by hydropower operations—on juvenile fish. Using a near-natural experimental facility (HyTEC), researchers conducted ~1,000 trials with >120,000 fish from four species (brown trout, European grayling, nase, and barbel) to understand how HPK intensity, environmental conditions, and biological factors influence stranding and downstream displacement.

Key results of the manuscript include:
• Hydropeaking intensity (ramping rate, peak duration, peak flow, number of peaks) is the dominant driver of fish stranding and displacement.
• Cold thermopeaking (rapid temperature drops during HPK) amplifies displacement.
• Nighttime conditions, small fish size, and low water temperatures significantly increase stranding risk.
• Behavioral adaptation: Displacement decreases after the first peak, suggesting learning, but stranding remains high.
• Species differences are minor; fish size matters more than species.
• Gravel bar slope and morphology influence outcomes—flat slopes increase stranding.

The study emphasizes that mitigation strategies should consider multiple factors beyond ramping rate, including time of day, fish size, and thermal changes.

Reply: Thank you for the positive evaluation of our manuscript!

Validity
I do not see any flaws that should prohibit the publication of this manuscript.

Reply: (No change necessary)

Originality and significance
Several of the results appear to be original to this manuscript, including the effects of environmental conditions (e.g., water temperature and diel period) on stranding and displacement. The paper also provides quantitative thresholds for ramping rate, temperature, and fish size that can inform mitigation

strategies. These results are of immediate relevance to fish ecologists, managers, dam owner/operators, and policy makers, and they extend to hydropower engineering, environmental regulation, and renewable energy planning, making the study significant across multiple disciplines.

Reply: (No change necessary)

Data & methodology

The approach is methodologically sound and well-aligned with the study objectives. The use of a near-natural experimental facility (HyTEC) with controlled hydropeaking scenarios provides ecological realism while maintaining experimental control. The large dataset—nearly 1,000 trials involving >120,000 fish—offers strong statistical power and species coverage across larval and juvenile stages. The experimental design effectively isolates hydropeaking effects by controlling confounding variables (e.g., temperature, morphology).

The data are extensive, covering multiple species, size classes, diel conditions, and hydropeaking intensities. Random forest modeling and SHAP analysis are appropriate for capturing non-linear relationships and interactions among multiple predictors. Threshold identification is supported by robust variance partitioning and cross-validation. Extended data tables and figures provide detailed variable importance, partial plots, and interaction analyses.

The methodology is described in detail, including experimental phases (stocking, acclimation, up-/down-ramping), facility specifications (channel dimensions, flow control, temperature mixing), and statistical procedures (SMOTE balancing, random forest parameters, SHAP interpretation). Extended Data and supplementary materials include threshold values, model performance metrics, and interaction plots. Data and code availability via Zenodo DOI ensures reproducibility.

Figures and tables are clear, with appropriate legends and references to Extended Data. Partial dependence plots and SHAP visualizations enhance interpretability. Some complexity in Extended Data may require careful reading, but overall presentation is strong.

Reply: (No change necessary)

Appropriate use of statistics and treatment of uncertainties

The study uses random forest models with cross-validation, variable importance metrics, and SHAP analysis to interpret predictor contributions. These methods are appropriate for handling complex, non-linear relationships and interactions in large datasets. The approach of using mean split values from tree levels and partial dependence plots is valid for identifying ecological thresholds. The application of SMOTE to address class imbalance is correctly implemented and described.

The main figures (e.g., variable importance, partial plots) do not display traditional error bars because they represent model-derived relationships rather than raw experimental means. Extended Data figures include partial dependence plots and SHAP values, which inherently convey uncertainty through model variance rather than confidence intervals. Probability values (p-values) are reported for correlation analyses (e.g., $r = 0.623$, $p < 0.001$), and these appear accurate and appropriately interpreted.

Reply: (No change necessary)

Conclusions

Overall, the conclusions and data interpretation appear robust and well-supported by the experimental design and statistical analysis. The large sample size (>120,000 fish across nearly 1,000 trials) and near-natural experimental conditions provide strong ecological realism and statistical power. The use of

advanced modeling techniques (random forests, SHAP) is appropriate for capturing complex, non-linear relationships and interactions among hydropeaking intensity, environmental factors, and biological traits.

Reply: (No change necessary)

Suggested improvements
I wonder whether the reduction in displacement after the first peak observed in treatments with multiple hydropeaking events, which the authors attributed to learning, might instead be caused by a change in the size distribution of remaining fish. The study acknowledges that stranding and displacement probabilities are strongly size-dependent—smaller fish (<17 mm) have roughly twice the stranding risk compared to larger individuals (>25 mm), and displacement also decreases slightly with increasing size. In treatments with multiple hydropeaking events, fish that survive the first peak are likely to be larger on average than those initially stocked, simply because smaller individuals are more prone to stranding or drifting out early. This could bias subsequent observations by artificially lowering stranding and drift rates in later peaks, making it appear that fish are "adapting" behaviorally when part of the effect may be due to a shift in size distribution. The authors interpret reduced displacement after the first peak as a learning effect, but they do not explicitly control for or quantify changes in size composition across consecutive peaks. Therefore, some of the observed adaptation could indeed be confounded by size-selective survival. This deserves either some level of discussion in the manuscript or additional data of how the size distribution changes over multiple peaking events (if available).

Reply: We clarified in the revised manuscript: Even though fish used in our multipeaking experiments had similar body lengths, size-selective effects in other contexts cannot be ruled out (see page 5 lines 20-21 and page 9 lines 4-6).

References
The manuscript generally references previous literature thoroughly and appropriately. It cites foundational work on hydropeaking impacts (e.g., Bradford et al., 1995; Halleraker et al., 2003), temperature effects (Donaldson et al., 2008), diel behavior (Bardonnet et al., 1991, 2006), and recent advances in ecohydraulics and thermopeaking (Auer et al., 2023; Hayes et al., 2019). The reference list is extensive and includes both classic studies and recent systematic reviews, which strengthens the context. Interestingly, a recent paper by Harnish et al. (2023) found that many of the same biological (i.e., fish size) and environmental factors (i.e., flow, temperature, diel period) that affect stranding and displacement also affect the probability of powerhouse entrainment. Harnish et al. (2023) attributed the observed effects to changes in the swimming ability and activity level of fish under varying conditions. The authors may consider reviewing that paper.

Harnish, R.A., K.D. Ham, J.R. Skalski, R.L. Townsend, and R.A. Buchanan. 2023. Factors affecting powerhouse passage of spring migrant smolts at federally operated hydroelectric dams of the Snake and Columbia rivers. Canadian Journal of Fisheries and Aquatic Sciences 80(12):1949-1966.

Reply: Thanks for this paper, which contains an impressive amount of data and analysis similar to ours. ;-) We think this reference supports our statement on p. 15 where we say that it is not enough to focus only on one parameter (like ramping rate) for successful mitigation, but we have to consider other biological and environmental factors (e.g., diel period, fish length, water temperature) to provide a complete, process-based understanding. We have added a sentence here referring to this reference.

Clarify and context
The abstract is generally clear and well-structured, effectively summarizing the research problem, methodology, key findings, and implications. It highlights the novelty and practical relevance of the study.

The introduction is comprehensive and appropriate, providing strong context, a thorough literature review, and clearly stated hypotheses. It logically progresses from identifying knowledge gaps to explaining the study design. The conclusions appropriately synthesize results and link them to management implications, stressing the need for multi-factor mitigation strategies. While practical recommendations are well articulated, the interpretation of behavioral adaptation (learning effect) is presented with confidence and would benefit from a cautionary note acknowledging potential confounding factors such as size-selective survival. Overall, these sections are well-developed and suitable for the intended audience.

Reply: We appreciate the positive summary. As suggested, we added a cautionary note about size-selective survival and other confounding factors in repeated exposure to hydropeaking (see page 9 lines 4-6.

Inflammatory material
The manuscript does not contain language that is inappropriate or potentially libelous.

Reply: Thanks for your constructive comments and the time and effort you put into reviewing this manuscript!

Reviewer #2 (Remarks to the Author):

The manuscript addresses an important and timely topic: the effects of hydropeaking on fish, with a particular focus on stranding/displacement processes. A major strength of the study is the exceptional experimental dataset, collected over an impressive 10-year period with extensive replication in a truly unique experimental facility. The work clearly builds on a set of prior publications derived from the same experimental system; these studies appear essential for context and interpretation and are appropriately cited.

Reply: Many thanks for the thoughtful review!

Overall, the manuscript is well written and I consider it suitable for publication after minor revision, mainly to improve structure, flow, and the breadth of interpretation. In its current (journal-driven) format, a large amount of key information (including all tables) appears in the Extended Data. Also for me non-traditional structure (apparently following journal format)where results are combined with a discussion results in too brief discussion, while broader interpretation remains rather limited. I recommend strengthening the discussion and embedding the findings in a wider ecological and life-history context. In particular, interspecific differences could be more clearly highlighted and interpreted, and the section headings and logical progression of the manuscript could be reconsidered to improve readability and narrative continuity.

Reply: Indeed, the current format is due to specifics of this journal, but we did move the main materials and methods from the supplementary information to the main text. We expanded the discussion on interspecific differences, integrating also a recent field study from the Ain River, France (see page 11, lines 42- page 12, lines 1-7). Also, we expanded the discussion to touch upon wider ecological and life-history contexts (e.g., page 13, lines 17-20).

A key point that should be clarified already at the outset is the scope of the study with respect to fish size and life stage. The work focuses on young/small fish (up to ~70 mm), which is well justified later in the text, but this should be stated explicitly in the title and/or abstract because it strongly conditions interpretation and generality. Related to this, while the manuscript argues convincingly that early post-

hatch stages experience a major bottleneck and are therefore especially relevant, it would be valuable to acknowledge more explicitly that hydropeaking-related disturbance of spawning may also lead to loss of eggs through displacement and subsequent mortality (e.g., drying or detachment from the substrate). These pre-hatch processes can also translate into substantial losses of potential recruits and deserve explicit mention to provide a more balanced life-stage framing.

Reply: Agreed! We now mention in the abstract and in the 'objective paragraph' of the introduction that the study deals with "larval and juvenile fish."

Thanks also for the comment on spawning and pre-hatch processes that can affect recruitment in hydropeaked rivers. We now integrated this into page 13, lines 17-20.

The introduction and presentation of the experimental facility could also be streamlined. The HyTEC facility is described well in detail in the Methods, but, a brief introduction of how it looks and work could be used early on, supported by an improved Figure 1: in addition to illustrating key river concepts and impacts, the figure could include a simple schematic of how those processes are represented in the experimental system (complementing the figure 1 in Extended Data).

Reply: Due to the complexity of the various experimental settings and the associated response of fish to hydropeaking, we decided to present the key elements in a conceptual figure and not within a natural setting, e.g., a photograph. This enabled us to visualize the effect of water level change in a three-dimensional perspective and to simultaneously incorporate the behavioral responses (movement of fish to the banks, drift, stranding, influence of morphology), while still keeping the principal spatial configuration of the experiments.

In terms of terminology, "ramping rate" does not need quotation marks; it should simply be defined once and then used consistently.

Reply: Agreed, we removed the quotation marks.

Where "thermopeaking" is mentioned, the expected impacts and relevance could be introduced more clearly.

Reply: We now mention thermopeaking already in the second paragraph of the introduction, stating that thermopeaking can exacerbate "hydropeaking-only" effects as the interactions are synergistic (see page 2, lines 15-17).

The final sentence of the first section of the main text appears to require a citation.

Reply: We added citations that underline that the mentioned factors, in addition to ramping rate, affect the response of fish to hydropeaking: Young et al. (2011), Moreira et al. (2019), Smokorowski (2021), and Hayes et al. (2022).

Species naming should also be consistent; grayling should be specified allways as European grayling.

Reply: We now always say "European grayling."

Several figure-related improvements would enhance accessibility. Figure 2 would benefit from using full variable names rather than codes (even if codes are defined in Extended Data), and the ordering could be adjusted so that the most important variables appear at the top for easier interpretation.

Reply: Done as suggested – see below.

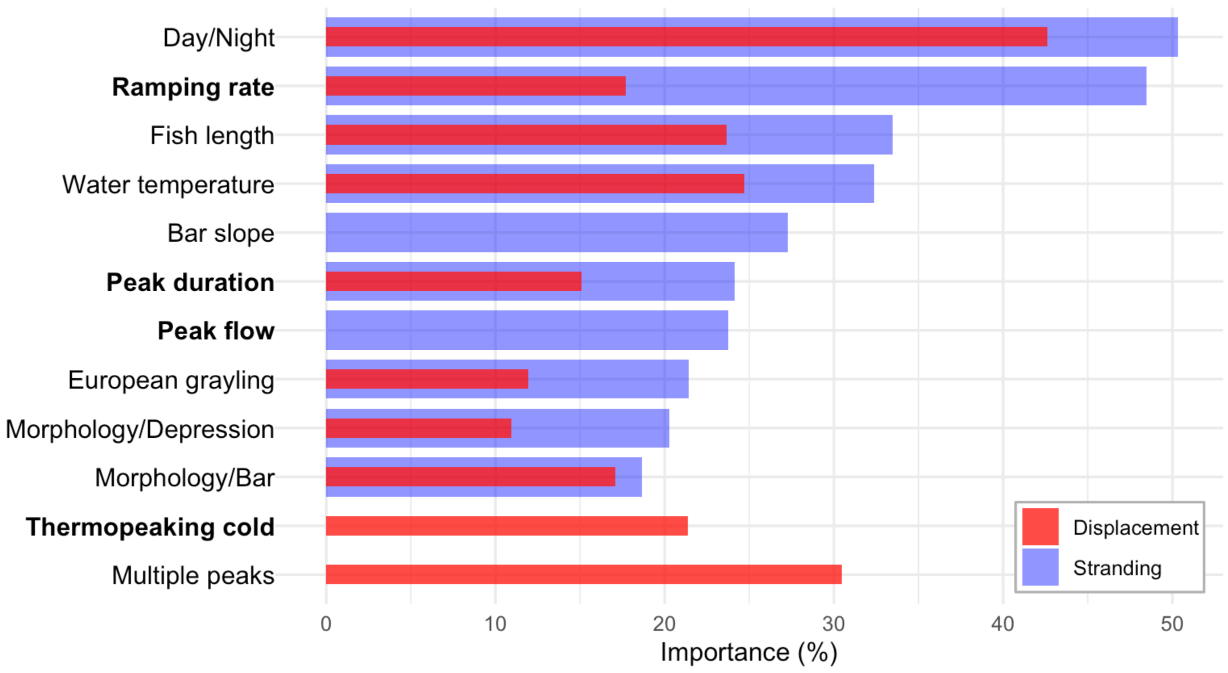

Peak flow should be reported in unit $m^3.s^{-1}$

Reply: We changed the flow units from l/s to $l.s^{-1}$ and from cm/min to $cm.min^{-1}$ throughout the manuscript.

(btw. why PeakFlow shows no importance for displacement infg 2?).

Reply: Peak flow is not among the 10 most important variables for displacement, only for stranding.

In the text, wording "Other HPK variables…" could be revised to "Besides ramping rate…" (or similar) to improve clarity.

Reply: We changed the sentence to: "Among hydropeaking variables, peak flow and peak duration were less influential than ramping rate in our stranding model, with thresholds of 100–130 $l.s^{-1}$ and 20 minutes, respectively"

In Figure 3, the visual hierarchy of labels could be improved, as "Hydropeaking variables" and "Stranding" currently appear at the same conceptual level (likewise for "other variables")

Reply: Done as suggested – see below.

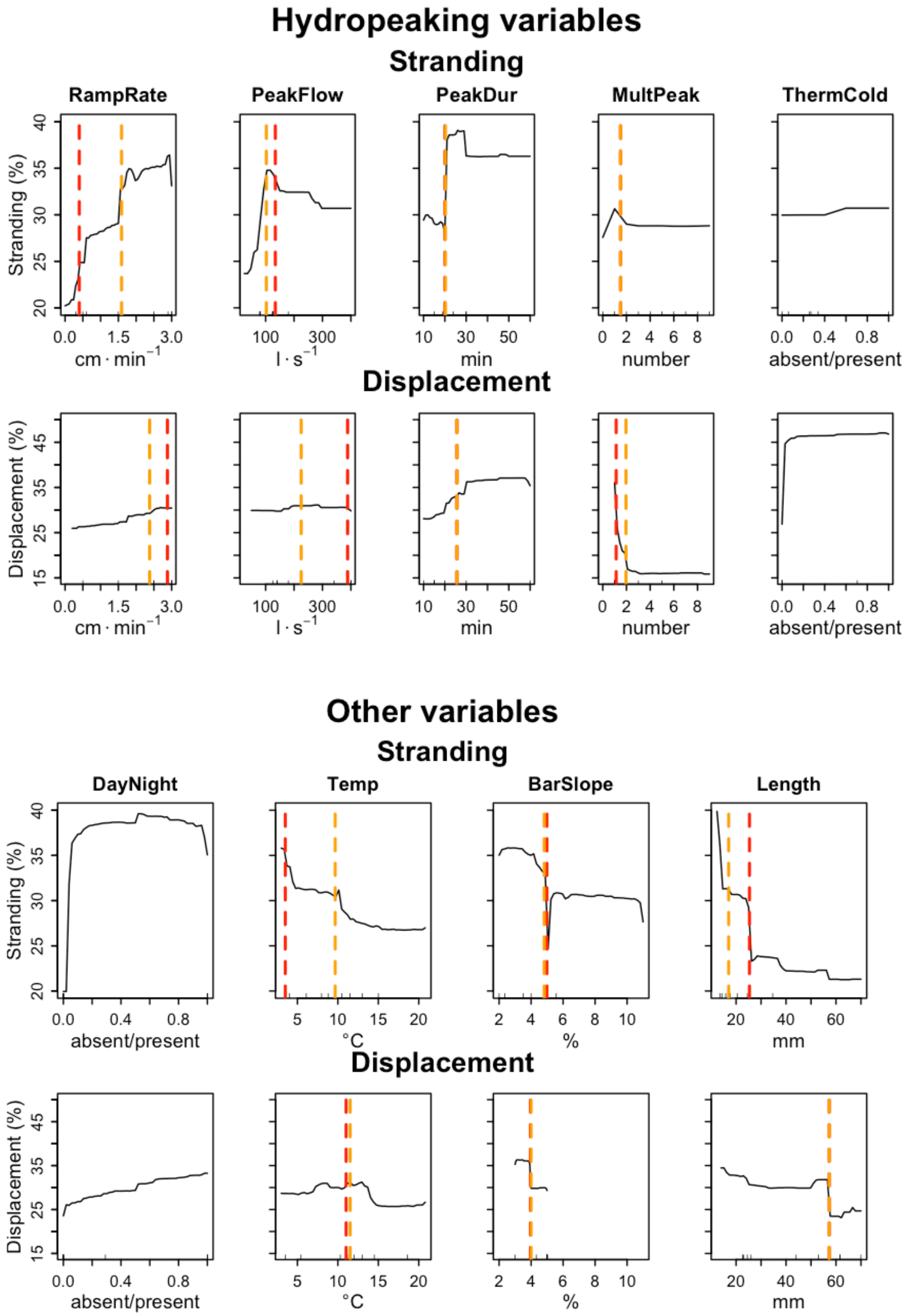

The Conclusions are clearly written and communicate the main outcomes effectively. However, given that the manuscript introduces five hypotheses at the beginning, the Conclusions (and ideally the abstract at a high level) should explicitly state whether each hypothesis is supported or not, so that readers can readily

extract the answers to the stated research questions. Finally, while the Methods are generally detailed and strong, they could be expanded with a concise description of the selected experimental species (key ecological traits, life-history considerations, and rationale for selection), which would also help interpret the interspecific patterns.

Reply: We now incorporate an evaluation of the hypotheses in the conclusions section:

We now include a detailed description of the key ecological traits and life-history considerations, on which we based the selection to use these four species of two fish families (see p. 5, lines 6-19).

In summary, this is a strong and valuable contribution based on a rare long-term, highly replicated experimental dataset. With minor revisions focused on clarifying scope (young/small fish), improving structure and narrative flow, strengthening discussion (especially regarding interspecific differences and broader context), explicitly answering the stated hypotheses, and making a few targeted improvements to figures and terminology, the manuscript should be well positioned for publication.

Reply: Thanks! As mentioned above, we clarified the scope, improved the narrative flow (esp. the methods), expanded the discussion, especially regarding interspecific differences and the broader context (e.g. spawning and pre-hatch effects on recruitment), and answer the stated hypothesis in the conclusions.

Reviewer #3 (Remarks to the Author):

Thanks for the opportunity to review this manuscript. The authors do a great job of explaining the relevance of the work and have an extremely large dataset collected in a purpose-built facility. The writing is very clear and concise until the methods section. I will acknowledge that I know very little about random forests, beyond it being a decision tree and machine learning method. I will defer to other reviewers on the specifics. However, I noticed some pretty glaring problems, either from incorrect use of the methods, lack of explanation, or lack of care/polish in the final product.

Reply: Thank you for taking the time to evaluate our manuscript and for providing helpful suggestions to improving the quality of the work!

First, I had a good idea of what the response variable was, but I had to look at the figure axis in one of the supplemental submissions to confirm you were using a percentage as the response. How was this determined? What was the experimental unit? I assume for each test, you recorded a single % for stranded/displaced, and this was the response. What about for the mesocosm experiments? Did you treat the 4 mesocosms as independent within a single test flow?

Reply: We agree that this explanation was lacking. We moved the material & methods part of the supplementary materials into the main text and expanded it to include a clear description of the response variable, stating that we used percentage (page 4, line 38); in this regard, we reference also Auer et al. (2017) who provide a detailed description of these calculations:

Table 2
Calculation of drift and stranding rates. R: amount of fish after adaption time; D_ur: amount of fish drifted out during upramping phase; D_p: amount of fish drifted out during peak phase; D_dr: amount of fish drifted out during downramping phase; C: residing fish removed during clearing phase; Σd_x: sum of fish drifted out during experimental phase (upramping-, peak- and downramping phase); Str: stranding.

Metric	Phase	Metrics	Calculation	
Drift	Upramping	D_ur	D_ur = d_ur / R * 100	Eq. (1)
Drift	Peak	D_p	D_p = d_p / (R − d_ur) * 100	Eq. (2)
Drift	Downramping	D_dr	D_dr = d_dr / (R − d_ur − d_p) * 100	Eq. (3)
Drift	Total	D_tot	D_tot = Σd_x / R * 100	Eq. (4)
Stranding	Downramping	Str	Str = (R − (Σd_x + C)) / (R − (d_ur + d_p)) * 100	Eq. (5)

Yes, each of the mesocosms was treated as an independent experiment within a single test flow (see Führer et al., 2022, 2024; Hayes et al., 2023). We now specify this in the supplementary materials.

How did you incorporate fish lengths, since those go to a single fish? Did you take the average length for a test flow? The descriptions of the data used in the study are lacking, even in the supplemental materials.

Reply: We now describe this in the methods section: "Fish lengths ranged from 12.4–70 mm, encompassing both larval and juvenile stages. Each experiment used fish of similar size; we therefore assigned the batch mean length to each experiment" (page 5, lines 19-21).

Many of the figures do not have adequate captions.

Reply: We checked the captions of all Figures and Tables and improved them where necessary. Particularly Figs. 4-5, Fig. S2, Figs. S6-9, and Table S4 were substantially revised.

The most concerning aspect is the figures themselves. There are several binary, categorical predictors (present/absent). The figure show the model estimating values in between, which don't exist. If there was a straight line between the means, sure, I can see what's going on, but many have non-linear relationships connecting absent to present. I don't know what this means, I don't know if this is a nuance of the model, I don't know if you've mis-specified the model. What it does mean is that nobody looked at the figures critically. Look at extended figure 7, barbel: it's a blank graph. What are we supposed to be learning from these? The lack of care in the presentation of the data does not give confidence for the amount of care put into the analysis.

Reply: The balancing procedure creates new samples also for binary data, resulting in values that are between 0 and 1. This is why there are also non-linear relationships calculated for the partial plots. For barbel no displacement experiments have been done. We included this updated figure (Fig. S7) in the final version and mentioned it in the figure caption.

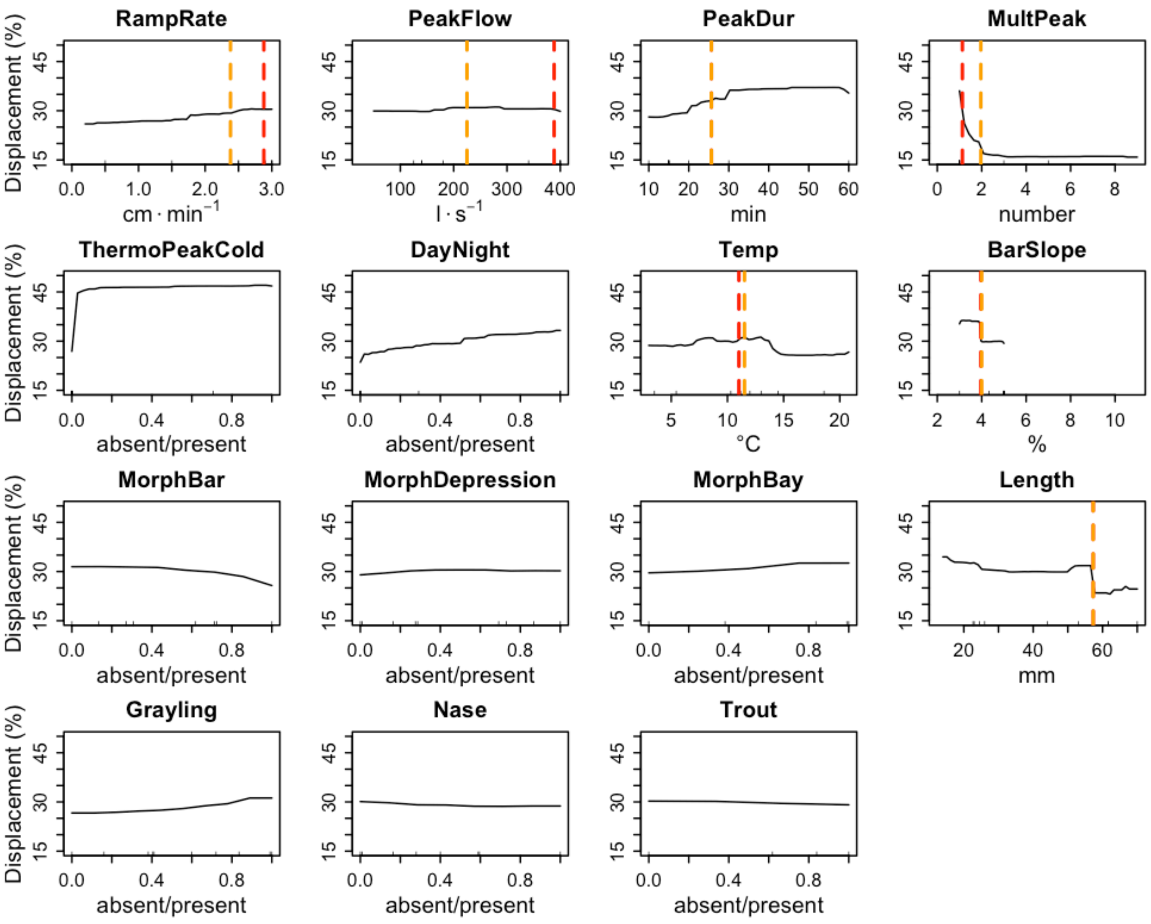

Fig. S7: Partial plots to visualize the partial dependence of the predicted displacement (%) on a specific predictor variable. Vertical lines indicate median split values for numeric variables extracted from random forest models (red: first tree level, orange: levels 2-5; see Table S5). Note: No displacement experiments were conducted for barbel; therefore, barbel is not shown.

Finally, I'm not sure what readers are supposed to learn from this. Ramping rate, as suspected, is the most important aspect of stranding and displacement. However, there's other factors, none of which are surprising at all. How does this get used? What are the recommendations to minimize the negative effects of hydropower? Using these results and applying them to a field study to show how the experimental study improves management of fish subjected to hydropeaking flow regimes would be widely applicable and useful for conservation. Here, you're just highlighting other factors that lead to stranding, none of which are surprising.

Reply: We appreciate the comment. Indeed, ramping rate has been the focus for a while. And while it is a dominant predictor, management decisions in hydropeaked systems are made under interacting constraints of other confounding parameters. Our contribution is to quantify how these co-drivers modulate stranding and displacement risk, and to translate that into decision-relevant guidance rather than focusing on ramping rate alone. Focusing solely on ramping rates risks incomplete or ineffective mitigation.

To aid management decisions, we place model results for stranding and displacement in the context of the most important variables (Figure 5): ramping rate, daytime, thermopeaking, and season (spring versus

winter). This scenario-based approach offers tangible recommendations for seasonal-dependent mitigation frameworks.

We further agree with the call for field validation, i.e., to implement these recommendations and study ecosystem responses in the field. We are currently conducting such studies with the aim to observe and quantify the responses of fish and macroinvertebrates with regard to reductions in hydropeaking frequency and ramping rate. Early results indicate improvements for some taxa but we expect more insights and ecosystem recovery in the next years.

References

Auer, S., Zeiringer, B., Führer, S., Tonolla, D. & Schmutz, S. Effects of river bank heterogeneity and time of day on drift and stranding of juvenile European grayling (Thymallus thymallus L.) caused by hydropeaking. *Sci. Total Environ.* 575, 1515–1521

Führer, S. *et al.* Variation in hydropeaking-induced stranding of Barbus barbus L. and Chondrostoma nasus L. larvae: Assessing the impact of daytime and down-ramping rates. *Ecohydrology* 17, (2024).

Führer, S. *et al.* Stranding of larval nase (Chondrostoma nasus L.) depending on bank slope, down-ramping rate and daytime. *Front. Environ. Sci.* 10, 1–18 (2022).

Hayes, D. S. *et al.* The interactive effect of river bank morphology and daytime on downstream displacement and stranding of cyprinid larvae in hydropeaking conditions. *Ecohydrol. Hydrobiol.* 23, 152–161 (2023).

Hayes, D. S. et al. Hydropeaking: Processes, Effects, and Mitigation. in Encyclopedia of Inland Waters, Second Edition 4, 134–149 (Elsevier Inc., 2022).

Moreira, M. et al. Ecologically-based criteria for hydropeaking mitigation: a review. Sci. Total Environ. 657, 1508–1522 (2019).

Smokorowski, K. E. The ups and downs of hydropeaking: a Canadian perspective on the need for, and ecological costs of, peaking hydropower production. Hydrobiologia 849, 421–441 (2022).

Young, P. S., Cech, J. J. & Thompson, L. C. Hydropower-related pulsed-flow impacts on stream fishes: A brief review, conceptual model, knowledge gaps, and research needs. Rev. Fish Biol. Fish. 21, 713–731 (2011).
